# Synergic mitigation of saline-alkaline stress in wheat plant by silicon and *Enterobacter* sp. FN0603

**DOI:** 10.3389/fmicb.2022.1100232

**Published:** 2023-01-16

**Authors:** Fangfang Xu, Yungang Liang, Xiaobing Wang, Yuze Guo, Kai Tang, Fuying Feng

**Affiliations:** ^1^Laboratory for Environmental Microbiology and Biotechnology in Arid and Cold Regions, College of Life Sciences, Inner Mongolia Agricultural University, Hohhot, China; ^2^Laboratory for Wheat Breeding and Cultivation, Institute of Crop Sciences, Inner Mongolia Academy of Agricultural and Animal Husbandry Sciences, Hohhot, China

**Keywords:** saline-alkaline, silicon, *Enterobacter*, root endophytic microorganism community, rhizosphere microorganism community, wheat

## Abstract

Although microorganisms and silicon are well documented as factors that mitigate salt stress, their effect mitigating saline-alkaline stress in plants remains unknown. In this study, wheat plant seeds were treated with silicon, *Enterobacter* sp. FN0603 alone and in combination of both. Wheat seeds were soaked in silicon and bacterial solutions and sown in pots containing artificial saline-alkaline soils to compare the effects among all treatments. The results showed that the treatments with silicon and FN0603 alone significantly changed plant morphology, enhanced the rhizosphere soil nutrient content and enzyme activities, improved some important antioxidant enzyme activities (e.g., superoxide dismutase) and the contents of small molecules (e.g., proline) that affected osmotic conditions in the top second leaves. However, treatment with silicon and FN0603 in combination significantly further increased these stress tolerance indexes and eventually promoted the plant growth dramatically compared to the treatments with silicon or FN0603 alone (*p* < 0.01), indicating a synergic plant growth-promoting effect. High relative abundance of strain FN0603 was detected in the treated plants roots, and silicon further improved the colonization of FN0603 in stressed wheat roots. Strain FN0603 particularly when present in combination with silicon changed the root endophytic bacterial and fungal communities rather than the rhizosphere communities. Bipartite network analysis, variation partitioning analysis and structure equation model further showed that strain FN0603 indirectly shaped root endophytic bacterial and fungal communities and improved plant physiology, rhizosphere soil properties and plant growth through significantly and positively directing FN0603-specific biomarkers (*p* < 0.05). This synergetic effect of silicon and plant growth-promoting microorganism in the mitigation of saline-alkaline stress in plants *via* shaping root endophyte community may provide a promising approach for sustainable agriculture in saline-alkaline soils.

## 1. Introduction

Soil salinization mainly occurs in semi-arid and arid area and influences 7% of the world’s filed. In soil salinization, neutral salts (mainly NaCl and Na_2_SO_4_) generate salt stress but alkali salts (mainly NaHCO_3_, Na_2_CO_3_) make additional alkali stress beside salt stress and lead to soil saline-alkalization (Wang et al., 2021). Neutral salt stress damages plants *via* water deficit within the plant, ion toxicity mainly Na^+^ and Cl^−^, nutritional imbalance resulting from reduction of nutrient uptake and/or transport to the shoot, accumulation of reactive oxygen species (ROS), and plasmolysis induced by ethylene signal (Mishra et al., 2013; Kerbab et al., 2021). Compared with neutral salts, alkali ones are more harmful to plants because of its high alkalinity which heavily reduce the nutrient availability. This leads to an imbalance of nutrients and ions in the plant (Guo et al., 2017; Song et al., 2021) and cause injury to photosynthetic systems characterized by chlorosis (Chen et al., 2011). Under such conditions, plant synthesize small molecules (e.g., proline and total soluble sugars) to maintain the osmotic balance and improve the activities of ROS scavenging enzymes (e.g., SOD, POD, and CAT) to reduce the oxidation of some key biomacromolecules such as nucleic acids, proteins and lipids (Hao et al., 2021). But till now, most studies used neutral salts to simulate saline-alkaline stress; only a few utilized alkaline salts to do that (Lu et al., 2022). Moreover, as neutral and alkali salts are usually co-existed, soil saline-alkalization influences a much larger land area. According to UNESCO (United Nations Educational, Scientific and Cultural Organization) and FAO (Food and Agriculture Organization), the saline-alkalization land area is more than 900 million hectares globally. One-third of global soil saline-alkalization occurs in China while about 70% of the Chinese occurs in semiarid and arid region of Northwest (Wang et al., 2021). Soil saline-alkalization of arable land will be increasing in the future if effective solutions are not applied (Fang et al., 2021; Kerbab et al., 2021). Therefore, there is a convergent and unmet need to discover strategies that migrate the saline-alkaline stress in plants in saline-alkaline soils.

Many studies have demonstrated that halotolerant, plant growth-promoting rhizobacteria (PGPR) can effectively eliminate salt stress, promote plant growth and enhance crop productivity under salt stress (Kumawat et al., 2022). Silicon is often used to enhance salt tolerance and promote plant growth (Mir et al., 2022) by various mechanisms like the positive regulation of phytohormones, reactive oxygen species-scavenging enzymes, osmotic-balancing solutes, plant photopigments and affecting other soil properties such as nutrient contents and enzyme activities (Kumawat et al., 2022; Mir et al., 2022). From approximately 2016 to the present, treatment with a combination of silicon and PGPR to effectively mitigate plant salt stress has attracted more attention due to its efficacy and an ecofriendly approach for future sustainable agriculture (Mahmood et al., 2016; Etesami and Adl, 2020; Kubi et al., 2021; Mahmood et al., 2022). Plant-microbe interactions are viewed as a key adaptive survival strategy in abiotic stress (Chen et al., 2022; Trivedi et al., 2022). But at present, the effect of PGPR on the microorganism community structure is not sufficient (Ji et al., 2021).

Wheat (*Triticum aestivum* L.) is one of most important cereal crops because it nourishes approximately 4.5 billion people, is planted extensively and has the potential for high global productivity (Mickky, 2022). Environmental stress is a factor in approximately 91% of global wheat cultivation, resulting in a 50% yield reduction (Kajla et al., 2015). Saline-alkalinity is a major environmental stress on crops cultivated in the northwest region of China (Zhang et al., 2021), which is the primary spring wheat-producing area. Treatment with silicon or PGPR alone could improve salt tolerance and promote the growth of wheat under neutral salt stress (Mickky, 2022), but the effects and mechanisms of a combination of silicon and PGPR on wheat under saline-alkaline stress is currently unknown.

The present study treated wheat seeds with silicon, a PGPR strain *Enterobacter* sp. FN0603 alone and in combination of both, employed NaCl and Na_2_CO_3_ to simulate saline-alkaline stress, mixed a handful of nature saline-alkaline soil as source to introduce halotolerant PGPR in the planting artificial soils, and determined and compared plant biomass, antioxidant enzyme activities, nutrient contents, microorganism community among the treatments, to (1) assess effects of silicon, *Enterobacter* sp. FN0603 and the combination of both on the wheat plant growth and physiobiochemical characteristics, rhizosphere soil physicochemical properties and bacterial and fungal community structures in rhizospheric soils and in roots and (2) elucidate the mechanisms of the saline-alkaline stress mitigation by silicon and the PGPR strain.

## 2. Materials and methods

### 2.1. Wheat cultivar and PGPR strain used and their basic traits

The wheat cultivar employed was Yongliang No. 4 spring wheat, which is widely planted in Northwest China where saline-alkaline stress reduces plant growth and limits crop production. Our previous experiments (Xu, 2017) showed that the germination rate of Yongliang No. 4 decreased to approximately 50% and seedling growth was significantly inhabited in the presence of 100 mM NaCl at pH 9.0.

Seeking salt-tolerant bacteria, we isolated PGPR from some typical desert plants such as *Zygophyllum xanthonylon* and *Haloxylon ammodendron* and tested their plant growth promoting properties, including nitrogen fixation, solubilization of inorganic phosphorus, siderophore production, 1-Aminocyclopropane-1-Carboxylate (ACC) deaminase activity, indole-3-acetic acid (IAA) production, biofilm formation and exopolysaccharide (EPS) production. A strain isolated from the rhizosphere soil of *Haloxylon ammodendron* (sampled from arid region of Northwest China; GPS location 107°54′22.6′′E, 42°15′41.8′′N), and designated FN0603, which displayed all of these plant growth-promoting traits and high salinity-alkalinity tolerance (Supplementary Table S1) and showed a remarkable growth-promoting potential for wheat under saline-alkaline stress when inoculated alone. A phylogenetic analysis indicated that this strain clustered with other *Enterobacter* and had the highest 16S rRNA gene sequence similarity (99.87%) with *Enterobacter hormaechei* EN-114^T^ (Supplementary Figure S1), suggesting that FN0603 belongs to the genus *Enterobacter*.

### 2.2. Seed treatments with silicon and FN0603

Four treatments were set up in this experiment, i.e., NU (null, no Si or FN treatments), Si (silicon-Na_2_SiO_3_ alone), FN (bacterial strain FN0603 alone) and FN_Si (a combination of silicon-Na_2_SiO_3_ and bacterial strain FN0603). Wheat seeds were treated by soaking them in the appropriate solution (i.e., sterilized 0.1% Carboxymethylcellulose sodium (CMC-Na) solutions containing silicon alone, FN0603 alone, both silicon and FN0603 or no silicon or FN0603) for 20 min. CMC-Na was employed to enhance adhesion to the seed surface.

An isolated single colony of FN0603 was inoculated into sterilized 1/2 R_2_A liquid medium (0.25 g peptone, 0.25 g casein hydrolysate, 0.25 g glucose, 0.25 g soluble starch, 0.15 g dipotassium phosphate, 0.012 g magnesium sulfate and 0.15 g sodium pyruvate in 1000 ml distilled water, pH 7.2), the culture was shaken with 160 rpm/min for 3 days then centrifugated to obtain the cells. The resulted cells were resuspended to an OD_600_ of 2.0 in sterilized 0.1% CMC-Na solution and immediately used to soak the seeds.

Healthy wheat seed grains were soaked in sterilized 0.1% CMC-Na solution for the NU treatment, in a sterilized 0.1% CMC-Na solution containing 0.8% Na_2_SiO_3_ for the Si treatment, in an FN0603 cell suspension for the FN treatment and in a mixture of Na_2_SiO_3_ and FN0603 (using the same number of FN0603 cells and the concentration of Na_2_SiO_3_ as in each individual solution) for the FN_Si treatment. After the excess liquid was removed, the seeds were immediately sown in pots.

### 2.3. Pot experiment

The surface soil (top 10 cm) of fields near Huhhot City (111°46′25.4”N, 40°38′29.8″E) and Barynoer City (40°58′37.4”N, 107°47′55.3″E) were collected as neutral bulk and saline-alkaline source soils, respectively. The collected soils were screened through a 2 mm sieve to remove debris such as roots and stone. The sieved bulk soils were mixed well with quartz sand in a 2:1 volume ratio and a small amount of sieved saline-alkaline source soils ( 1/100 of the bulk and quartz mixture volume) was placed in black plastic pots (16 × 14 cm) and irrigated with Hoagland’s solution ( 1/4 ×concentration of Hoagland solution adjusted the Na^+^ concentration to 100 mM and the pH to 9.0 with Na_2_CO_3_ and NaCl: firstly adjusted pH to 9.0 by Na_2_CO_3_, then calculated Na^+^ concentration and replenished by NaCl to 100 mM), until the volume escaping from the bottom of the pot did not change within 2 h. The resulting soil was considered completely saturated by the saline-alkaline solution. After the first irrigation, 10 seed grains were sowed in each pot and covered with 1 cm of the same soil as in the pot. Twenty replicate pots for each treatment were prepared. The pots were cultivated under conditions of 5,000 Lux light-intensity, a light/dark cycle of 14/10 h and a temperature of 25 ± 2°C in a room without sunlight. During cultivation, pots were regularly irrigated with 185 mL of sterile distilled water per pot when the leaves appeared to be wilting. Water overflew from pot bottom was poured back into the corresponding pot the next day to ensure a stable salt content. The saline-alkaline fields planted wheat probably enriched halotolerant microorganisms and they may interact with silicon and strain FN0603 to rapidly help wheat in responding the stress. The saline-alkaline fields are far away from our laboratory and moreover the soils are just needed minor as source. Thus, the pot experiment was designed to add minor saline-alkaline source soils.

### 2.4. Sampling of plants, rhizosphere soils and roots

Wheat plants of similar size and appearance (e.g., similar shoot height) were selected at 30 days after emerged, the entire plant was sampled, and the corresponding rhizosphere soil, leaves and roots were taken for further analysis. After the roots were carefully dug out, large soil chunks were removed by shaking and the residual soil brushed off, then the roots were quickly frozen in liquid nitrogen and stored at −80°C until ready for the rhizosphere soil analysis (Mendes et al., 2014). After the rhizosphere soil was removed, the roots were washed with sterile water for 30 s, then soaked in 70% ethanol for 2 min, followed by a soak in 2.5% NaClO containing 0.1% Tween 80 for 5 min, subsequently transferred to 70% sterile ethanol for 30 s, and finally washed with sterile water three times. The clean roots were quick-frozen with liquid nitrogen and then stored at −80°C for the root endophytic microorganism analysis (Ren et al., 2019). Five biological replicates were employed for all the samples, respectively.

### 2.5. Assays of wheat growth parameters

Wheat growth parameters including morphology, seedling emergence, tiller number, shoot length, root length, shoot dry weight and root dry weight were observed and measured. Germination rate was determined by the formula (number of seeds geminated/total number of seeds sown) described by Aniat et al. (2010). The growth morphology and tiller number of wheat were recorded when and after sampled. Length of shoot and root were measured using measuring scale in centimeter. After oven-drying at 60°C for 72 h, the shoots and roots were weighed for the dry weight. Based on biomass (i.e., the dry weight of the whole plant), salt tolerance indexes (STI) were calculated as according to Kumawat et al. (2018) using the Equation (1):


(1) $$\mathrm{STI}=\frac{\mathrm{TDW}\;\mathrm{at}\;\mathrm{one}\;\mathrm{of the salinity level}\;}{\mathrm{TDW}\;\mathrm{at}\;\mathrm{control}}\times 100,$$

where TDW- total (shoot + root) dry weight.

### 2.6. Biochemical analysis of wheat

The top second functional leaves were used for the determination of leaf protein, the proline and malondialdehyde (MDA) contents and the activities of the reactive oxygen species-scavenging enzymes superoxide dismutase (SOD), catalase (CAT) and peroxidase (POD). For chlorophyll and carotenoids analysis, 0.5 g fresh leaves were homogenized in 50 mL ethanol to extract pigments and determined OD_649_, OD_665,_ and OD_470_, respectively, and calculated using the Equations (Lichtenthaler and Wellburn, 1983) (2), (3), (4) and (5):


(2) $$\begin{array}{l} \mathrm{chlorophyll}\;a,C_{a}\left(\mathrm{mg}/g\right)\\ =\left(13.95\;{\mathrm{OD}}_{665}-6.88\;{\mathrm{OD}}_{649}\right)\times \left(V/1000\times W\right), \end{array}$$

(3) $$\begin{array}{l} \mathrm{chlorophyll}\;b,\;\;C_{b}\left(\mathrm{mg}/g\right)\\ =\left(24.96\;{\mathrm{OD}}_{649}-7.32\;{\mathrm{OD}}_{665}\right)\times \left(V/1000\times W\right), \end{array}$$

(4) $$\mathrm{total chlorophyll},\;\;C_{a+b}\left(\mathrm{mg}/g\right)=C_{a}+C_{b},$$

(5) $$\begin{array}{l} \mathrm{carotenoids},\;\;C_{c}\left(\mathrm{mg}/g\right)\\ =\left(\frac{1000\times {\mathrm{OD}}_{470}-2.05C_{a}-114.8C_{b}}{245}\right)\times \left(V/1000\times W\right), \end{array}$$

where V- the final volume of the supernatant and W- fresh weight of the leaf.

Fresh leaves of 0.1 g were crushed homogenously in 1 mL the particular extract liquid, and then determined using spectrometer methods followed the protocols of manufacturer (Suzhou Grace Biotechnology Co., Ltd.). The used *kits* were G0101F, G0105F, G0107F, G0109F, and G0124F for SOD, CAT, POD, MDA and root activity, respectively.

### 2.7. Assays of rhizosphere soil properties

Rhizosphere soil was air-dried and screened through 1 mm, then determined properties following the method according to Zhang et al. (2007). The contents of organic matter (OM) were determined using the K_2_Cr_2_O_7_-H_2_SO_4_ volumetric dilution heating method. Available nitrogen (AN) was determined by the alkaline diffusion method. Available phosphorus (AP) was extracted with 0.5 M NaHCO_3_ (pH 8.5) and measured at 880 nm. Rhizosphere soil and distilled water (1:5, m/v) were mixed and shaken for 30 min, then read the electrical conductivity (EC) of the filtered leach solution using conductivity meter. Soil and distilled water (1:10, m/v) were mixed and shaken for 30 min, then measured the pH of the filtered leach solution using pH meter.

For soil enzyme activity assays, rhizosphere soil was air-dried and screened at 60 mesh. Following the particular protocols, the activities of soil alcalase protease (ALPT), alkaline phosphatase (ALP), urease (UE), sucrase (SC), catalase (CAT) and soil dehydrogenase (DHA) were determined using Soil properties *kits* of Suzhou Grace Biotechnology Co., Ltd. The used *kits* were G0314W, G0305F, G0301W, G0302W, G0303W and G0307F for ALPT, ALP, UE, SC, CAT and DHA, respectively.

### 2.8. Analysis of the diversity and community structure of rhizosphere and root endophytic microorganism

According to the manufacturer’s protocols, DNA was extracted from the rhizosphere soil and the clean roots using the DNeasy^®^ PowerSoil^®^ Pro *Kit* (QIAGEN, NA, United States) and the FastDNA^®^ Spin *Kit* for Soil (MP Biomedicals, CA, United States), respectively. The fungal ITS1 regions were amplified by PCR using primers ITS1F (CTTGGTCATTTAGAGGAAGTAA) and ITS2R (GCTGCGTTCTTCATCGATGC). The V5–V7 regions of the root endophytic bacterial 16S ribosomal RNA gene were amplified by PCR using the primer pair 799F (AACMGGATTAGATACCCKG) and 1193R (ACGTCATCCCCACCTTCC), the V3-V4 regions of the rhizosphere bacterial 16S ribosomal RNA gene were amplified by PCR using the 338F primer (ACTCCTACGGGAGGCAGCAG) and the 806R primer (GGACTACHVGGGTWTCTAAT) by an ABI GeneAmp^®^ 9700 PCR thermocycler (ABI, CA, United States). The concentration and purity of the DNA was assessed using a NanoDrop^®^ ND-2000 spectrophotometer (Thermo Scientific Inc., MA, United States). A NEXTFLEX^®^ Rapid DNA-Seq *Kit* (Bioo Scientific, TE, United States) was used to build a library and Illumina’s Miseq PE300 platform (Illumina, SD, United States) was used for high-throughput sequencing. All amplicon sequencing was performed by Shanghai Majorbio Bio-pharm Technology Co., Ltd. (Shanghai, China).

The Fastp software^1^ (Chen et al., 2018) was used for quality control of the original sequence, the and the FLASH software^2^ (Tanja and Steven, 2011) for mosaic analysis. Based on the default parameters, the DADA2 (Callahan et al., 2016) plug-in in the Qiime2 process (Bolyen et al., 2019) was used to de-noise the optimized sequences after quality control splicing to get the amplicon sequence variants (ASVs) sequence. The chloroplast and mitochondrial sequences annotated in all samples were removed, and the number of sequences in all samples was equalized to 20,000. The Good’s coverage of each sample could reach 99.09%. A species taxonomic analysis of ASVs was performed using the Naive Bayes classifier from Qiime2 based on the Silva (Ver. 138)16S rRNA gene database and Unite (Ver. 8.0) ITS gene database. The *α*- and *β*- diversity indices were calculated as described by Tang et al. (2021).

### 2.9. Identification of specific biomarkers

A linear discriminant analysis (LDA) effect size (LEfSe) analysis (Segata et al., 2011)^3^ was used to identify the biomarkers present in the various treatments (LDA > 3, *p* < 0.05) that had significant differences in abundance. Referred to Wang et al. (2022), differential genera between four treatments were divided into three groups, namely Group Si, Group FN and Group FN_Si. For instance, differential genera between Si and NU treatments were combined with differential genera between FN_Si and FN treatments into a group named by Group Si and defined as the group’s biomarkers (further split into and labeled as rhizo_biomarkers_Si and endo_biomarkers_Si corresponding to rhizospheric and root endophytic microorganisms, respectively). Similarly, the differential genera between FN and NU were combined with between FN_Si and Si in Group FN; ones between FN_Si and NU was in Group FN_Si; the obtained differential genera in the individual groups were defined as the groups’ biomarkers. Distance matrixes for soil properties, wheat physiology, wheat growth and the root-microorganism communities were calculated with a principal coordinate (PCoA) dimensionality reduction analysis using the R package of the Vegan software (ver. 2.5-7). The values of the PCo1 axis and PCo2 axis were compared with the values before dimensionality reduction by a Spearman correlation (|*r*| > 0.6, *p* < 0.05) using the R package “psych” (Revelle and Revelle, 2015). The axis which showed the higher significance correlation was used as the representative axis for the subsequent analysis (shown in yellow mark in Supplementary Data Sheet 1).

### 2.10. Mantel test and bipartite network analysis

A Mantel test (based on Bray–Curtis) of the physiologic properties of the wheat and soil and all of the root-associate microbiome was conducted using the R package “linkET” (Li T. et al., 2022) after scaling and calculating the distance matrices. A bipartite network analysis was also conducted to assess the relationships among microorganism communities for genus, soil properties, wheat physiology and wheat growth. The network was generated using the R package “ggClusterNet” (Wen et al., 2022) and visualized by Gephi v0.9.271 (Bastian et al., 2009).

### 2.11. Construction of structural equation model

The results of the Mantel test and network indicated that most of soil properties, wheat physiology and wheat growth parameters were poorly correlated with the rhizosphere communities, so the structural equation model (SEM) and variation partitioning analysis (VPA) were only focused on the root endophytic communities.

A SEM was implemented with the software IBM SPSS AMOS (Ver. 23) with maximum-likelihood estimation and visualized by the affinity designer software (Serif Ltd., Nottingham, United Kingdom). Model fitness was examined using the *χ*^2^ tests, the comparative fit index (CFI) and the root mean square error of approximation (RMSEA). Low *χ*^2^ values (*p* > 0.05), high CFI (>0.90) and low RMSEA (<0.05) indicated a well-fitting model. VPA determined the explanatory degree among strain FN0603, specific biomarkers, root endophytic bacterial communities, root endophytic fungal communities, rhizosphere soil properties, wheat physiology and wheat growth.

### 2.12. Statistical analysis

The statistical analysis of the data was carried out with a one-way analysis of variance (ANOVA) and the results were considered significant at the levels of *p* < 0.05 or *p* < 0.01 and labeled with uppercase or lowercase letters, respectively. Column diagrams of the physiologic parameters of the wheat and soil were drawn with the R package “EasyStat” (Zhu et al., 2022).

## 3. Results

### 3.1. Effects of silicon and *Enterobacter* sp. FN0603 on the growth parameters and physiology of wheat under saline-alkaline stress

**Figure 1:**
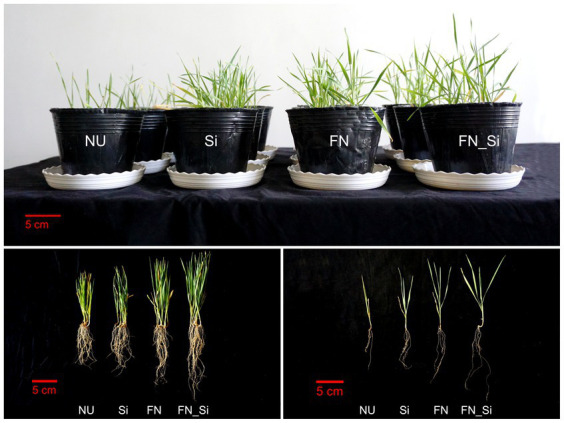
Morphology of wheat under salt-alkaline stress.

**Figure 2:**
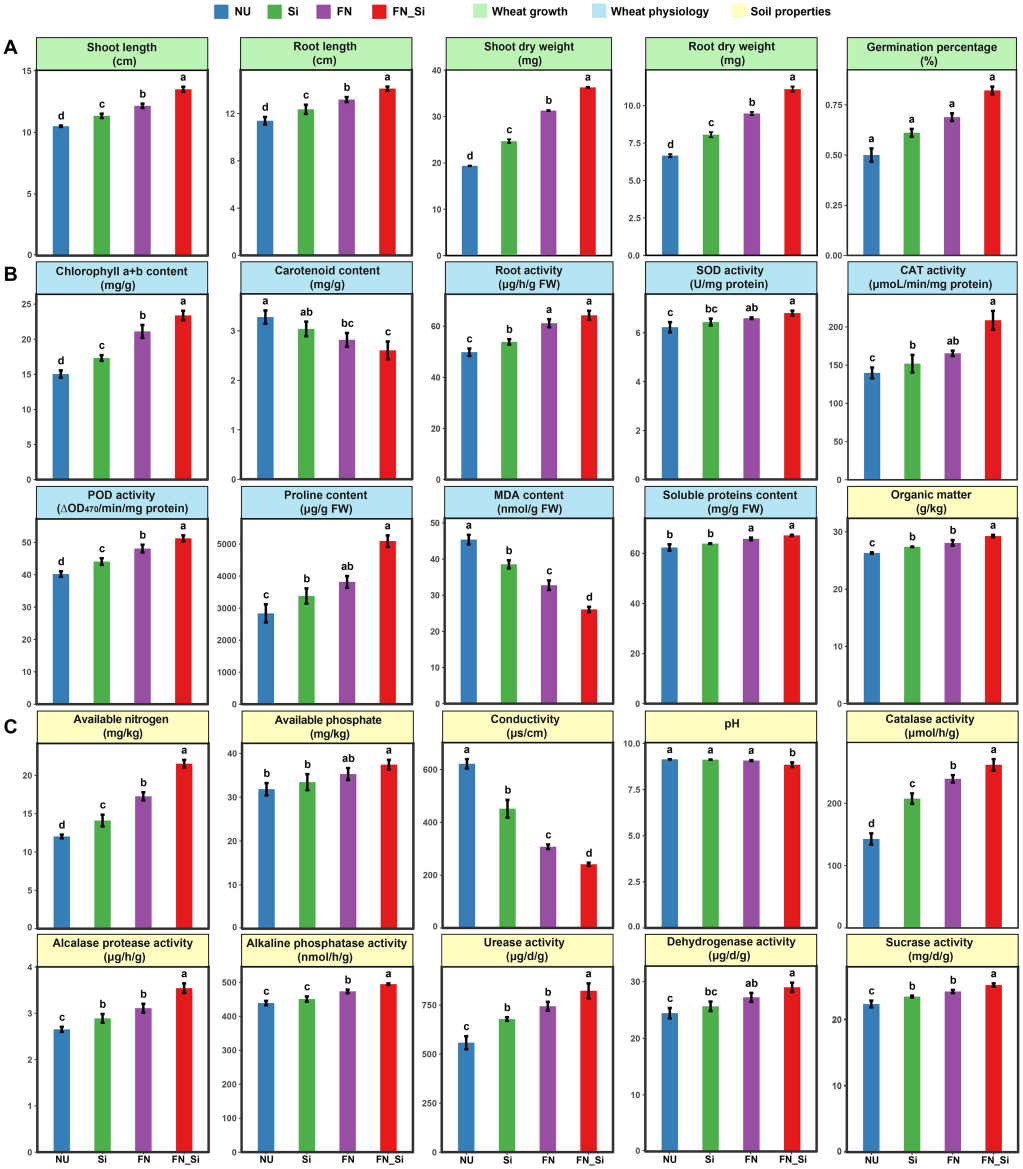
**(A)** Wheat growth, **(B)** wheat physiology, and **(C)** soil properties. Error bars indicate the SD from the mean among treatments. Letters indicate significant differences at *p* < 0.05.

Thirty days after emergence, several commonly used plant growth parameters were analyzed. Compared with NU, the Si and FN treatments both evidenced remarkably different morphologies (Figure 1), with the significant increases of the shoot and root lengths of wheat under saline-alkaline stress (Figure 2A). However, in the FN_Si treatment, all plant growth parameters were significantly higher than Si and FN treatments (Figure 2A). For example, the shoot and root dry weight for FN_Si were 1.9 and 1.7, 1.5, and 1.4 and 1.2 and 1.2 times greater than NU, Si and FN, respectively. Moreover, FN_Si also showed extremely and significantly higher tiller numbers, 7.5, 3 and 2.5 times greater than NU, Si and FN, respectively (Supplementary Table S2). Ultimately, the improved plant growth parameters can be ascribed to the salt tolerance index for the wheat under stress as affected by the treatments (Supplementary Table S3). Compared with Si and FN, the index for FN_Si, respectively improved to 144.75 and 116.32. Additionally, FN0603 alone highly significantly enhanced all growth parameters (e.g., root dry weight, 1.2 times compared to Si), implying that treatment with *Enterobacter* strain FN0603 is more effective than with silicon. Obviously, both silicon and FN0603 can promote wheat growth under saline-alkaline stress conditions but the presence of both more effectively mitigates the stress.

Physiological parameters of the functional wheat leaves were determined, including the antioxidase activities, chlorophyll content, carotenoid content and root activities, which are commonly responsible for the plant antioxidant capacity, as well as the proline and the total soluble protein contents, which indicate the osmotic regulation capacity under salt stress (Figure 2B). The results showed that all of the enzyme activities increased with treatment, with the largest increase of 49.25% of CAT activity occurring in FN_Si compared to NU. Treatment with either one or a combination of Si and FN elevated the total soluble protein and proline contents but decreased the carotenoid and MDA levels with highly significant differences (*p* < 0.01). The contents of the pigment chlorophyll and the root activities were significantly enhanced in Si, FN and FN_Si compared to NU as well. The magnitude of the effects followed the order FN_Si, FN and Si.

### 3.2. Effects of silicon and *Enterobacter* sp. FN0603 on rhizosphere soil properties of wheat under saline-alkaline stress

Significant differences in the improvements of the physiochemical parameters and enzyme activities of the rhizosphere soil were observed in the presence of silicon and FN0603 alone as well as in the combination treatment compared to NU (Figure 2C). In major nutrients (i.e., OM, AN, and AP), the most improvement occurred in AN, and FN_Si, FN and Si was 79.02, 52.64 and 24.70% higher than NU, respectively; while the most improvement for soil enzymes (i.e., CAT, ALPT, ALP, UE, DHA, and SC) was occurred in CAT, and FN_Si, FN and Si was 83.31, 26.13, and 9.37% higher than NU, respectively. Nevertheless, EC and pH were both decreased in all treatments compared to NU with the greatest effect in FN_Si, which showed decreases of 61.2, 46.6, and 21.7% for salinity and 3.21, 3, and 2.5% for pH compared with NU, FN and Si, respectively. All differences were extremely significant (*p* < 0.01).

### 3.3. Effects of silicon and *Enterobacter* sp. FN0603 on the diversity and composition of root-associated microorganism community of wheat under saline-alkaline stress

Bacterial and fungal *α*-diversity box plots showed that the Chao1 and Shannon indexes were approximately one order of magnitude greater for rhizosphere microorganisms than for the corresponding root endophytes; nevertheless the *α*-diversity of the root endophytes was much higher for the treatments compared to the rhizosphere microorganisms. The treatments did not show striking differences in the Chao1 and Shannon indexes of the rhizosphere bacteria, but a significant increase in the Chao1 index of the rhizosphere fungi was seen, particularly for FN_Si, which was 10.43% higher compared to NU (Supplementary Figure S2A). Silicon alone significantly increased the Chao1 indexes (*p* < 0.05) of the root endophytic bacteria and fungi; Silicon and FN0603 alone reduced the Shannon indexes of the root endophytic bacteria and fungi, and the combination extremely significantly (*p* < 0.01) decreased the Shannon indexes up to 42.5 and 42.3%, respectively (Supplementary Figure S2B).

A PCoA was employed to evaluate the *β*-diversity. The results showed that the treatments, especially the combination of silicon and FN0603, showed a *β*-diversity well-separated from the other treatments, indicating significant changes in the rhizosphere and root endophytic bacterial and fungal community structures (Adonis test; Supplementary Figure S3).

**Figure 3:**
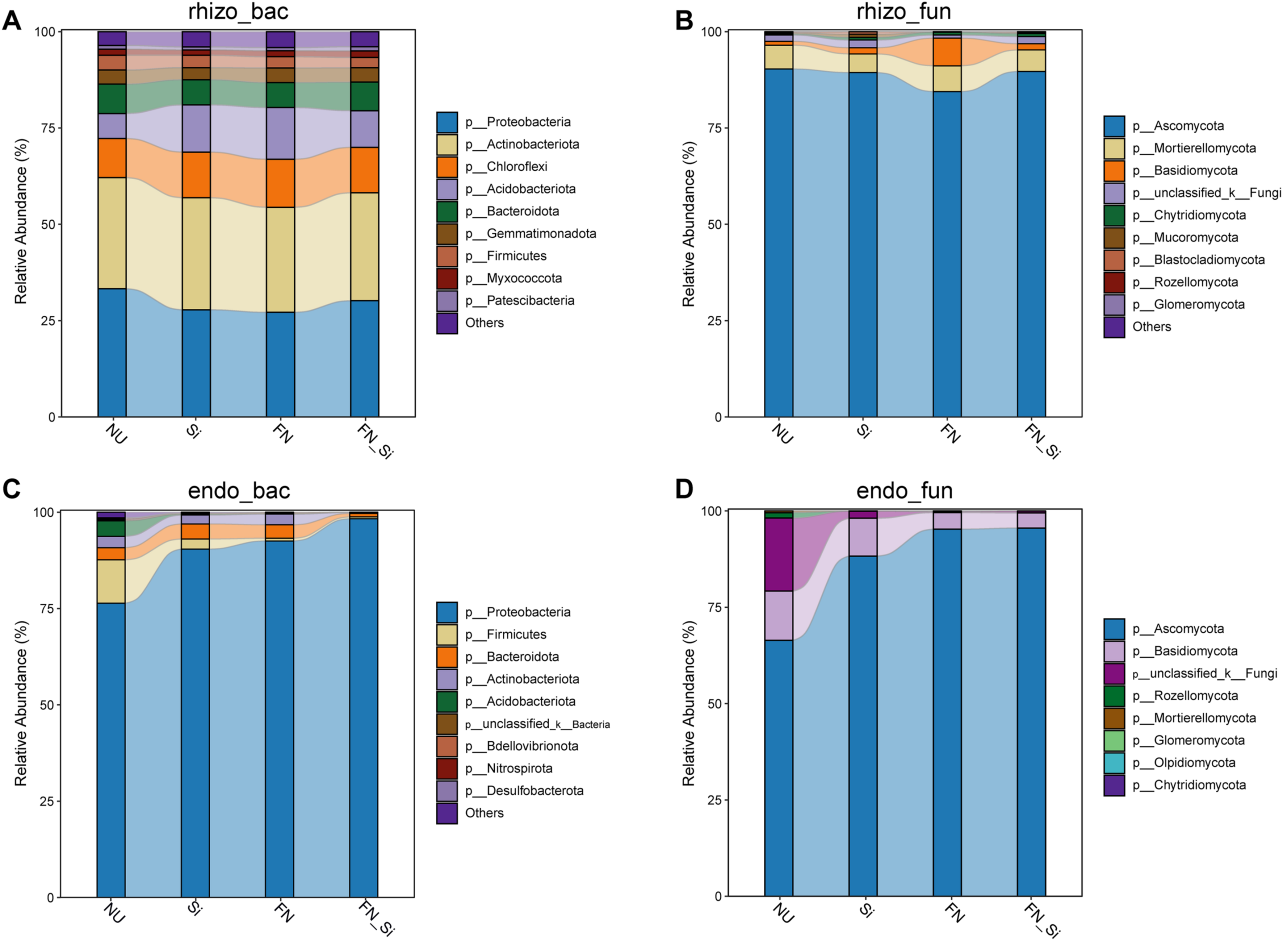
**(A)** Rhizosphere bacterial community, **(B)** rhizosphere fungal community, **(C)** root endophytic bacterial community, and **(D)** root endophytic fungal community composition at phylum level.

A total of nine predominant bacterial phyla were identified from rhizosphere bacterial community, including Proteobacteria, Actinobacteriota, Chloroflexi, Acidobacteriota, Bacteroidota, Gemmatimonadota, Firmicutes, Myxococcota and Patescibacteria, among which Proteobacteria (27.22–33.27%) and Actinobacteriota (27.28–28.92%) were greatest (Figure 3A). Only five predominant phyla, i.e., Proteobacteria, Firmicutes, Bacteroidota, Actinobacteriota and Acidobacteriota were found for the root endophytic bacteria, Proteobacteria accounted for 76.43–98.31%, and was the most predominant root endophytic bacterium (Figure 3C). For all treatments, the relative abundance of Chloroflexi and Acidobacteriota was markedly increased, whereas Proteobacteria and Firmicutes decreased for the rhizosphere bacteria. Predominant genera (i.e., a relative abundance greater than 5%) were *Arthrobacter* (8.29–11.05%), *Pseudomonas* (2.68–4.56%) and *Enterobacter* (0.18–1.09%) for the rhizosphere bacterial community (Supplementary Figure S4A). For the root endophytic bacterial community, the treatments markedly increased the relative abundance of Proteobacteria but decreased the relative abundance of Firmicutes, Actinobacteriota and Acidobacteriota. The genus *Enterobacter* (4.12–47.95%) was dominant and its abundance increased with treatment in the order Si, FN and FN_Si (Supplementary Figure S4C). Additionally, FN_Si remarkably increased the abundance of *Pseudomonas* to 21.70%.

For the rhizosphere fungal community, four predominant phyla were seen, including Ascomycota, Mortierellomycota, Basidiomycota and one unknown phylum, among which Ascomycota was the most dominant with an abundance of 84.50–90.41% (Figure 3B). In the root endophytic fungal community, the treatments, particularly the combination, further significantly increased the abundance of Ascomycota and decreased Basidiomycota and one unknown phylum (Figure 3D). At the genus level in the rhizosphere fungal community, the treatments, particularly FN_Si, further significantly increased the abundance of *Neocosmospora* and *Fusicolla*, and decreased *Fusarium* (Supplementary Figure S4B). For root endophytic fungal community, all treatments gradually increased the abundance of genus *Fusarium* up to 70.51% from 6.61% in NU; in contrast, *Apiotrichum* decreased with treatment from 10.33% in NU to 3.18% in FN_Si (Supplementary Figure S4D).

The sequences of ASV5038 and ASV1 had 100% similarity with the corresponding partial 16S rRNA gene of strain FN0603 retrieved from the rhizosphere and root endophytic high-throughput sequencing data (Supplementary Figure S5). A much lower abundance ASV5038 was detected in the rhizosphere high-throughput sequencing data from all four treatments; even the inoculation with FN0603 decreased the abundance, and the decrease in FN_Si was significantly different (Supplementary Figure S6A). Similarly, for the root endophytic data, FN and FN_Si showed a remarkably increased the abundance of ASV1 approximately one order of magnitude greater compared to the other two treatments, and FN _Si was significantly greater than FN alone (respectively 3.7, 3.0, 27.9 and 36.0% for NU, Si, FN and FN_Si; *p* < 0.01; Supplementary Figure S6B). These results indicated that FN0603 could colonize wheat roots; the strain is an endophyte and colonization is further improved in the presence of silicon.

### 3.4. Biomarkers of root-associated microorganism of wheat under saline-alkaline stress

For the rhizosphere microorganisms, the numbers of biomarkers were higher in Group Si (12 bacteria and 5 fungi general) and FN (17 and 5) than in Group FN_Si (7 and 3); but for the root endophytes, the numbers were greater in Group FN_Si (43 bacteria and 5 fungi general) and FN (39 and 13) than in Si (35 and 3) (Supplementary Figures S7–S10). A correlation analysis further showed that root endophytic bacteria and fungi biomarkers of 18 in Group FN and 20 in Group FN_Si were significantly related to FN0603 (*p* < 0.05), respectively; and they included 14 genera shared by the two groups. Among 14 genera, the relative abundances of four genera (two bacterial and two fungal), *Enterobacter*, one unclassified (belong to Order Enterobacterales), *Fusarium*, and *Myrmecridium*, were significantly increased in FN_Si over Si and FN treatments, suggesting that the four genera with high abundance may have a stronger interaction between strain FN0603 along with silicon and wheat plant under saline-alkaline stress.

### 3.5. Correlations among strain FN0603, FN0603-specific biomarkers, microorganism communities, rhizosphere soil properties, and wheat physiology of wheat under saline-alkaline stress

**Figure 4:**
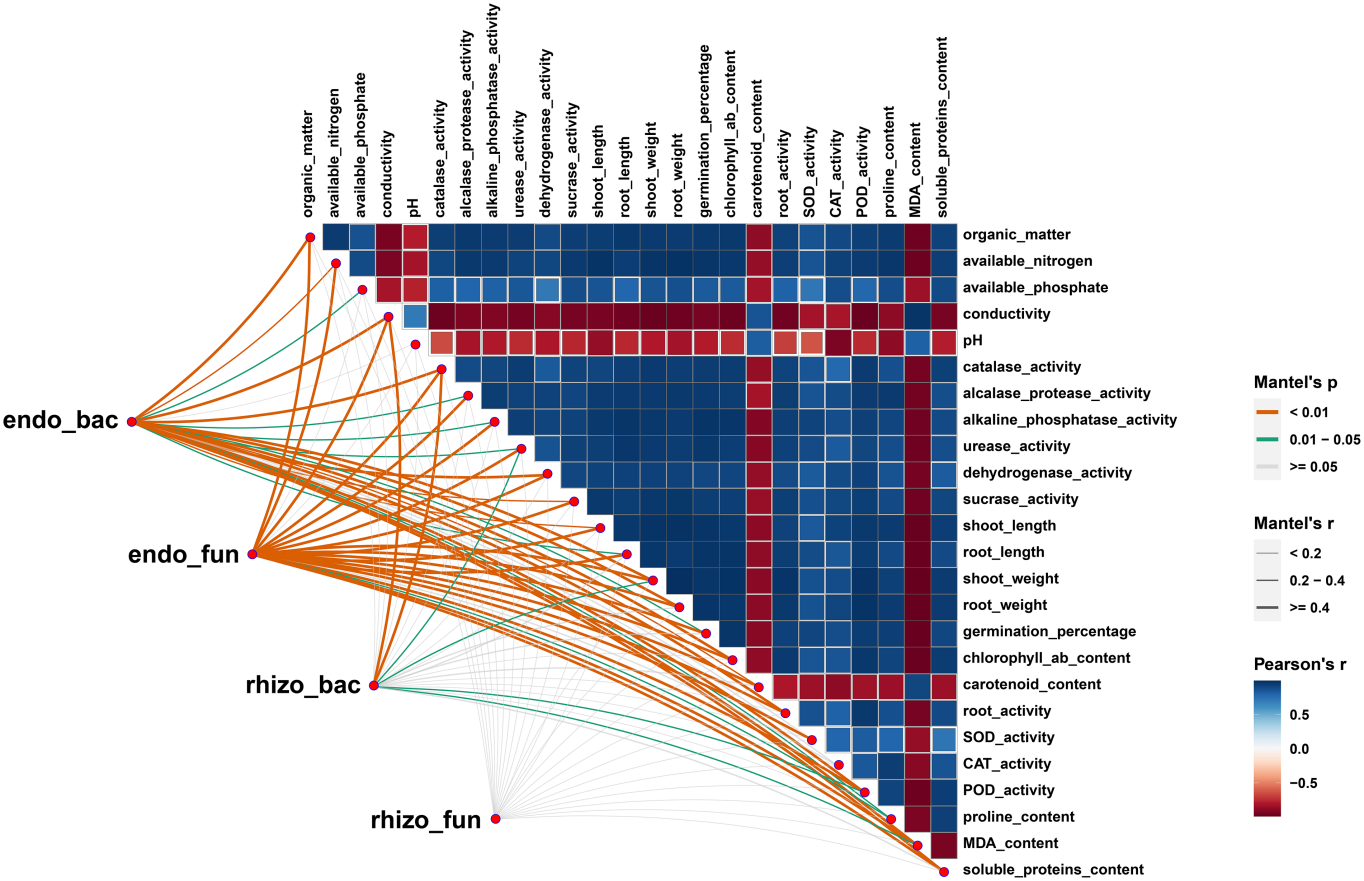
Mantel test showed the correlation among root-associate microorganisms, wheat physiology and soil properties. The size of square represents Pearson Correlation Coefficient (*r*) among different physiology properties factors.

**Figure 5:**
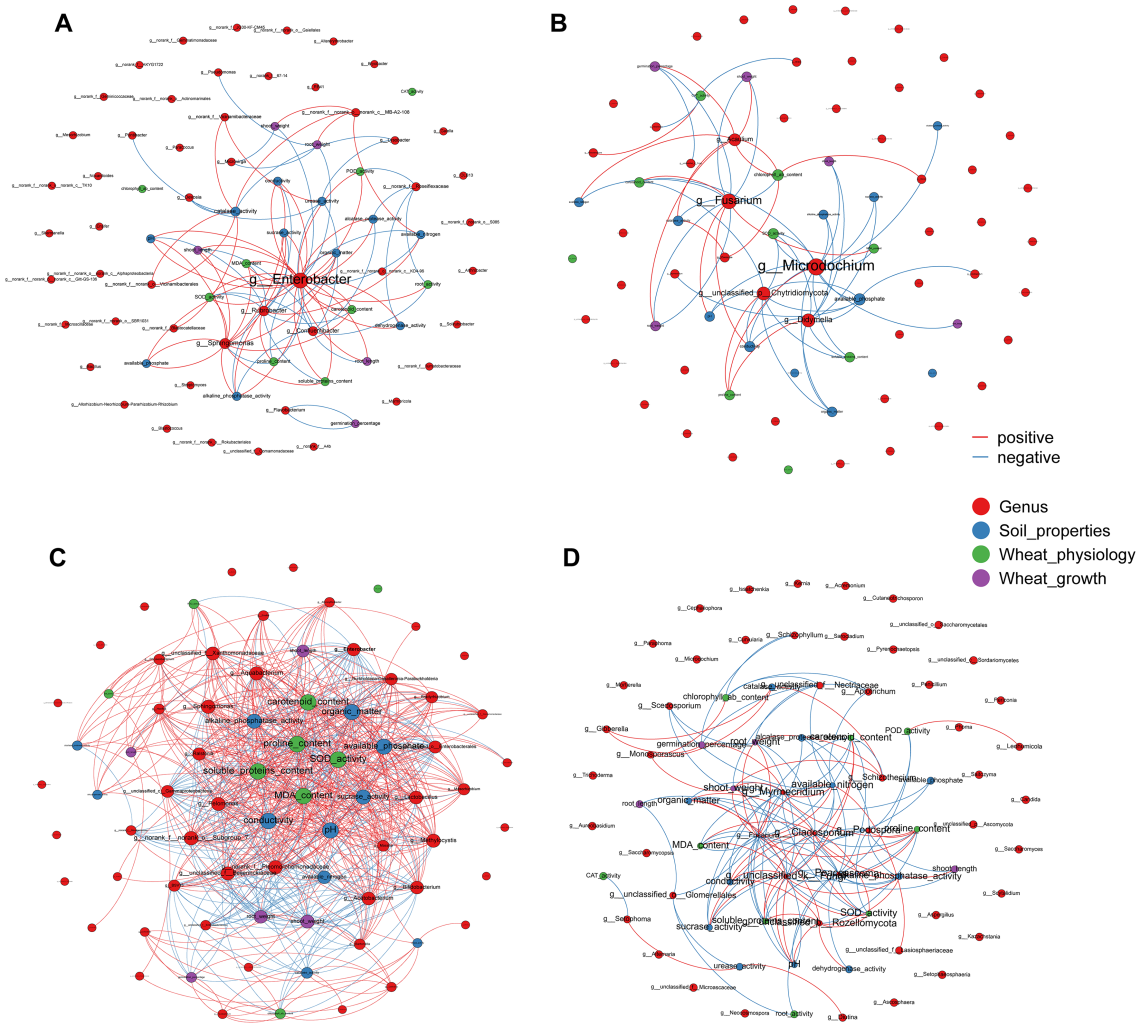
Network analysis depicting an interaction pattern among generals of **(A)** rhizosphere bacterial communities, **(B)** rhizosphere fungal communities, **(C)** root endophytic bacterial communities, and **(D)** root endophytic fungal communities, wheat physiology, soil properties and wheat growth. The size of nodes and labels represent the degree of connection. The red line indicated the positive interaction and blue line indicated negative interaction. The top 50 species with|*r*| > 0.6 and *p* < 0.05 were retained.

**Figure 6:**
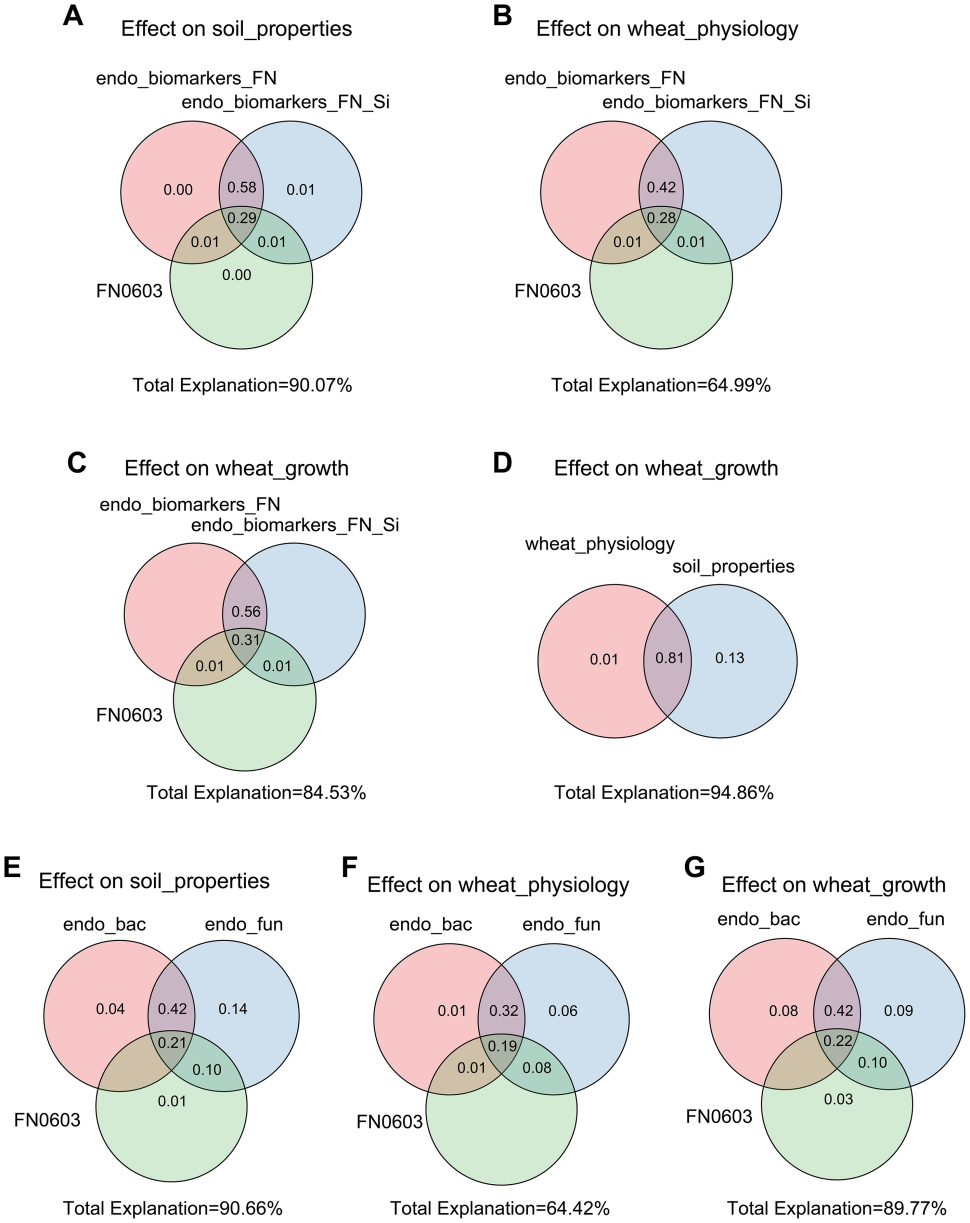
Variation partitioning analysis (VPA) evaluated the explanatory degree of FN0603, endo_biomarkers_FN and endo_biomarkers_FN_Si to the variation of **(A)** soil properties, **(B)** wheat physiology and **(C)** wheat growth. The explanatory degree of soil properties and wheat physiology to the variation of **(D)** wheat growth. The explanatory degree of FN0603, root endophytic bacterial communities and root endophytic fungal communities to the variation of **(E)** soil properties, **(F)** wheat physiology, and **(G)** wheat growth. All the specific biomarkers were FN0603-specific biomarkers.

The Mantel tests revealed significant positive correlations between rhizosphere soil properties, wheat physiology and wheat growth (*p* < 0.01), but EC, pH and MDA had negative effects on these parameters (Figure 4). Rhizosphere microorganisms were not significantly correlated with major soil properties, or wheat plant physiology. But in striking contrast, root endophytic microorganism communities showed extremely significant correlations with the majority (*p* < 0.01) and significant correlations with a few soil properties, wheat plant physiology and growth (*p* < 0.05; Figure 4). Consistent with this, the network based on the Spearman correlation assay also indicated that the endophytic microorganism communities were significantly correlated with the rhizosphere soil properties, wheat physiology and wheat growth. These included *Enterobacter*, unclassified_f_*Enterobacteriaceae*, unclassified_o_*Enterobacterales*, *Aquabacterium*, *Sphingomonas* and *Pelomonas* from the endophytic bacterial community and *Fusarium*, *Poaceascoma* and *Myrmecridium* genera from the endophytic fungal community (Figures 5C,D). Inconsistently with the Mantel tests, the Spearman correlation indicated that *Enterobacter, Sphingomonas* and *Rubrobacter* from the rhizosphere bacterial community were significantly and positively correlated with almost all wheat plant physiology, soil properties and wheat growth. Moreover, *Microdochium* and *Fusarium* from the rhizosphere fungal community were significantly affected wheat and soil properties (Figures 5A,B). Consistently, the VPA further indicated that the highest total explanatory power for wheat growth were wheat physiology and rhizosphere soil properties (81.98 and 94.05%, respectively) (Figure 6D); the highest total explanatory power for rhizosphere soil properties (90.66%) were FN0603, endophytic bacterial community and endophytic fungal community (Figure 6E); the highest total explanatory power for wheat physiology were FN0603, endo_biomarkers_FN and endo_biomarkers_FN_Si (64.99%) (Figure 6B); for the endophytic bacterial community and endophytic fungal community were FN0603, endo_biomarkers_FN and endo_biomarkers_FN_Si (39.74 and 16.06% respectively) (Supplementary Figure S11).

### 3.6. Effects of strain FN0603 on microorganism communities, biomarkers, rhizosphere soil properties, and wheat physiology under saline-alkaline stress

**Figure 7:**
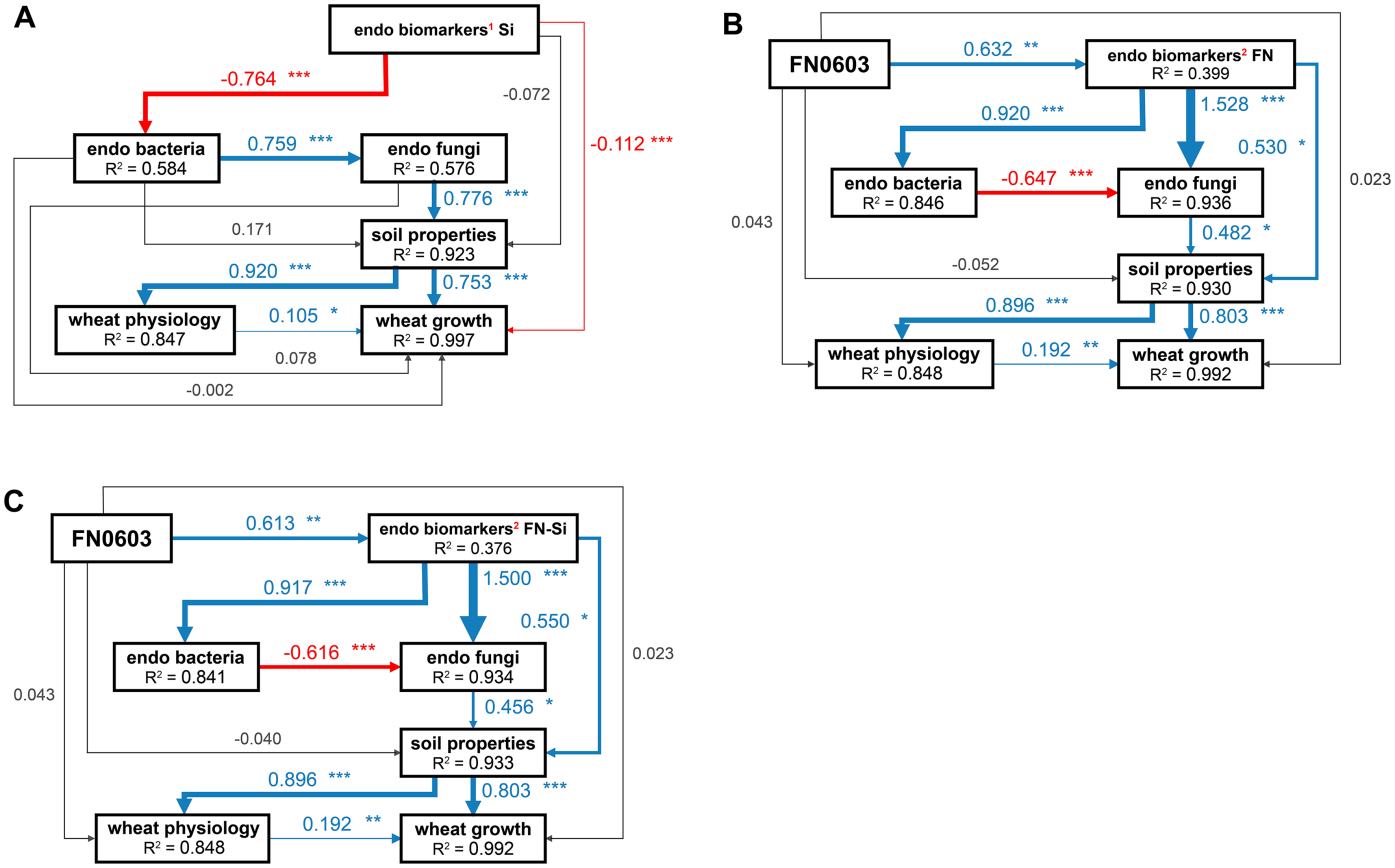
Structural equation model (SEM) analyzing the directly and indirectly effect paths of **(A)** Si, **(B)** FN and **(C)** FN_Si on wheat growth through specific biomarkers, root endophytic microorganisms, soil properties, and wheat physiology. Numbers adjacent to each arrow are the standardized path coefficients. The width of each arrow is proportional to the strength of its corresponding path coefficient. *R*^2^ denotes the proportion of variance explained. Blue and red arrows indicate positive and negative relationships, gray line indicate non-significant relationships, respectively. * Represents a significant difference (***p < 0.001; **p < 0.01; *p < 0.05), ^1^represents not FN-specific biomarkers, ^2^represents FN-specific biomarkers.

SEM was employed to evaluate the direct and indirect effects of treatment with silicon alone, FN0603 alone and silicon and FN0603 combined on the microbial communities, soil properties, wheat physiology, and wheat growth. In all groups defined by the LEfSe assay above, all of the models fit the data well. Wheat growth was directly, positively and significantly affected by rhizosphere soil properties (λ of 0.753–0.803, *p* < 0.001) and wheat physiology (λ of 0.105–0.192, *p* < 0.05); soil properties were directly and positively affected by the root endophytic fungal community (λ of 0.456–0.776; highly significantly, *p* < 0.001 in Group Si and *p* < 0.05 in the other two groups) and bacterial communities (not significant) (Figures 7A–C). Silicon alone affected the endophytic bacterial community, producing significant, direct and positive effects on the fungal community (*λ* = 0.759, *p* < 0.001) (Group Si shown in Figure 7A), whereas FN0603 alone (Group FN shown in Figure 7B; *λ* = −0.647) and the combination of FN0603 and silicon (Group FN_Si shown in Figure 7C; *λ* = −0.616) showed endophytic bacterial community have an significant and direct but negative effects on the endophytic fungal community (*p* < 0.001).

In Group Si, endophytic biomarkers (composed of bacteria and fungi, the same as in the following) had significant, direct and negative effects on the endophytic bacterial community (*λ* = −0.764, *p* < 0.001), but the endophytic biomarkers induced by and significantly correlated with FN0603 had extremely significant, direct and positive effects on the endophytic bacterial (*λ* = 0.920 and 0.917 in Group FN and Group FN_Si, respectively; *p* < 0.001) and fungal communities (*λ* = 1.528 and 1.500 in Group FN and Group FN-Si, respectively; *p* < 0.001). FN0603 directly affected endophytic biomarkers in Group FN (*λ* = 0.632, *p* < 0.01) and Group FN_Si (*λ* = 0.613, *p* < 0.01). Besides indirect effects through mediating the entophytic bacterial and fungal communities, the significant and positive direct effects of endophytic biomarkers on soil properties were observed with λ values of 0.530 and 0.550 (*p* < 0.05) in Group FN and Group FN_Si, respectively. The models of Group Si, FN and FN-Si explained 37.6–58.4% of the endophytic biomarkers of the variation in the root endophytic bacteria and 84.8–99.7% of the variation in the other items.

## 4. Discussion

High salinity and alkalinity greatly limit plant growth and production, especially when the adverse alkaline conditions are caused by Na_2_CO_3_ or NaHCO_3_ and occur during the initial phase of the plant life cycle (e.g., seedling; Liu et al., 2022; Pérez-Rodriguez et al., 2022). Chemical treatment of seeds with silicon or inoculation with a biological agent such as PGPR could eliminate salt damage to the plants (Kubi et al., 2021), but the effects of treating seeds with a combination of silicon and PGPR on plants under saline-alkaline stress are less clear. This study first showed significant effects on the growth wheat seedlings when the seeds are treated with silicon and PGPR and indicated that the mechanism was related to plant morphology and physiology, rhizosphere soil properties and particularly to root-associated microorganisms.

Morphological, physiological and biochemical traits are commonly used as indicators for plant health and growth, since alterations in these parameters could provide important adaption information for plants subjected to the combined stress of salt and alkali (An et al., 2021). Treatment with silicon or PGPR alone could counteract salt stress damage and change morphological and physio-biochemical characteristics, such as significant increases in shoot and root lengths, fresh and dry weight, chlorophyll content (Chakraborti et al., 2022; Ellouzi et al., 2022; Herms et al., 2022; Mickky, 2022; Mir et al., 2022). Although more serious stress is present with high alkali caused by CO_3_^2−^ in addition to Na^+^, treatment with silicon or *Enterobacter* sp. FN0603 alone also led to similar protection of wheat in this study. However, treatment with a combination of silicon and PGPR had extremely significant protective effects on roots, shoots, biomass and chlorophyll, evidencing further improvement of wheat salt tolerance compared to the impact of silicon and *Enterobacter* sp. FN0603 alone. Moreover, the combination increased the tiller numbers of wheat compared to untreated and singly treated plants, showing highly significant differences. Tiller number increases were observed when nitrogen and phosphorus fertilizer were applied (Saber et al., 2012) and some PGPR such as *Enterobacter* sp. inoculated (Nadeem et al., 2013; Wang et al., 2020) when wheat was planted in saline soil. Exogenous hormones increased tiller number in wheat and greatly affected yield (Cai et al., 2014). FN0603 is a strong plant hormone (e.g., IAA) producer and silicon may increase that capability. Our recent field data (not shown) indicated that PGPR inoculation promoted successful tiller development and ultimately enhanced wheat yield. Therefore, these observations suggested that increased soil nutrient availability and hormones produced by bacteria and PGPR-stimulated plants may have contributed to the tiller number increase, ultimately resulting in a higher yield.

Abiotic stresses such as salt and alkali lead to reactive oxygen accumulation and plant damage (Lu et al., 2022; Ren et al., 2022). The inoculation of PGPR improves the expression and activity of reactive oxygen species-scavenging enzymes and proline production, which balances osmotic pressure, but MDA produced from membrane oxidation decreases in wheat under saline and/or alkaline stress (Haroon et al., 2022; Li F. et al., 2022). Similarly, this study showed that treatment with silicon or *Enterobacter* sp. FN0603, compared to an untreated control showed significantly (*p* < 0.05) enhanced activities of the reactive oxygen species-scavenging enzymes SOD, CAT and POD, and increased the proline content and reduced the MDA content in the wheat leaves in the presence of saline-alkaline stress. However, treatment with a combination of silicon and FN0603 effectively improved the antioxidant capability by further increasing the activities of the reactive oxygen species-scavenging enzymes and the proline content and decreasing the MDA content, compared to treatment with either silicon or FN0603 alone, the differences were highly significant (*p* < 0.01). Unexpectedly however, the leaf carotenoids level of treated wheat plants under saline-alkaline stress were lower, and the combination treatment even reduced those levels by 20.5% compared to the untreated control in this study. This result was distinctly different from the common finding that carotenoids are significantly increased under salt stress (Haroon et al., 2022), suggesting that the protective mechanism for saline-alkaline stress differed from that for salt stress. The decease of carotenoids probably due to the increase of the abundance of *Fusarium* and *Myrmecridium*, two fungal genera of strain FN0603-specific biomarker in root endophytes, for which significantly and negatively correlated with carotenoids content (Figure 5) in current study.

Seed-priming by inoculation with PGPR activates the rhizosphere soil by improving nutrient levels, such as available nitrogen and phosphorus and organic matter, as well as the activities of soil enzymes (Chakraborti et al., 2022; Mickky, 2022). These observations were consistent with our findings in this study. However, the combination still significantly improved the effects of these factors compared to treatment with silicon or FN0603—for instance, available nitrogen increased to 21.52 mg/kg from 12.02 mg/kg in the control, 14.10 mg/kg with silicon alone and 17.26 mg/kg with *Enterobacter* sp. FN0603 alone. *Enterobacter* sp. FN0603 stimulated multiple plant growth-promoting factors such as ACC-deaminase, siderophore production, IAA-production, nitrogen fixation, and P-Ca_3_(PO_4_)_2_ solubilization, which positively affected the nutrient cycle and may have importantly contributed to the protection of wheat under saline-alkaline stress. These observations suggest that the interaction between silicon and FN0603 is synergistic and the combining silicon and PGPR treatments is a more effective approach to mitigate saline-alkaline stress in plants.

Halotolerant *Enterobacter* mitigates salt stress and promote plant growth by various processes, such as the production of ACC deaminase, IAA biosynthesis, phosphorus solubilization, nitrogen fixation, siderophore production, EPS secretion and biofilm formation (Etesami and Glick, 2020). *Enterobacter* was one of core endophytic microbiota in many plants (Riva et al., 2022) and particular important to the salt tolerance of wheat (Chen et al., 2022). The *Enterobacter* sp. FN0603 used in this study encompasses many of these growth-promoting traits and is a multiple growth-promoting bacterium. Identical sequences were found both in rhizosphere soil and root endophytic bacterial high-throughput sequencing data based on an ASV analysis, indicating that *Enterobacter* sp. FN0603 colonized the wheat rhizosphere and the roots (Supplementary Figures S5, S6), although it was isolated from the rhizospheric soils of desert plant. It is generally realized that most endophytic microorganisms are derived from soil and optional, coming from soil and colonizing in plant tissues after selecting by plant and competing with other microbiota (Santos and Olivares, 2021). Some important endophytic microbiotas would be enriched with increasing the relative abundance responded to environmental stress (Santos and Olivares, 2021; Zhang et al., 2022). Treating seeds with FN0603 failed to markedly increase the strain’s relative abundance and distinctly change the microorganism community structure in the rhizosphere soil, but it significantly enhanced the strain’s relative abundance and changed the microorganism community structure in root endophytes. Particularly when combined with silicon, the abundance of the strain FN0603 was significantly maximized compared with treatment with silicon alone (*p* < 0.01), although the solely inoculated of silicon did not affect the abundance of the strain FN0603, suggesting that silicon promoted the colonization of FN0603 in wheat roots under saline-alkaline stress. As far as we know, this is the first report of such a function for silicon. According to the abundance-occupancy concept (Shade and Stopnisek, 2019), consistently occurred and even with high abundance in case of the bioinoculated, strain FN0603 would be one of core endophytic microbiota recruited and enriched to encounter saline-alkaline stress by wheat.

Our study demonstrated that there were little effects on the diversity and structure of microorganism community in rhizospheric soil, but remarkable alterations occurred to root endophytic microorganism by treated with strain FN0603 and the combination of silicon and strain FN0603 (Supplementary Figure S2; Figure 3). The treatments, particular the combination of silicon and strain FN0603 decreased root endophytic bacterial diversity and richness but increased those of fungal community. This is similar with the effect of endophytic *Bacillus* sp. on rhizospheric and root endophytic microorganism community structure of wheat under salt stress (Ji et al., 2021). As to community structure, the combination treatment significantly improved the abundance of *Pseudomonas* and reduced that of *Ralstonia*; the former is a PGPR genus regularly used for alleviating abiotic stress (Chakraborti et al., 2022) and the latter is well known as a plant pathogen (Denny, 2007). Consistent with our findings, inoculation with other beneficial microorganisms associated with wheat plant growth promotion also changed the root endophytic bacteria community. For instance, one remarkable change was a significant improvement of *Pseudomonas* in wheat root after inoculated with arbuscular mycorrhizal fungus *Funneliformis mosseae* IMA1 and the wheat root endophytic bacterium *Lactobacillus plantarum* (Agnolucci et al., 2019). Even in the natural simulative microorganism community assembly, bacteria family Enterobacteriaceae and Pseudomonadaceae, among which composed of *Enterobacter* and *Pseudomonas* respectively, was found positively linked and predominated in root endophytes (Goldford et al., 2018). Tremendous colonization of *Pantoea* sp. in rice root were driven by salt stress (Bhise and Dandge, 2019). These suggested that plant may employ recruitment of endophytes to defense against saline-alkaline stress. However, results that plants belonging to *Curcurbitaceae* recruited rhizosphere rather than root endophytic bacteria to adapt to salt stress under natural conditions, inconsistently with our findings (Li et al., 2021). For the root endophytic fungal communities, all treatments (i.e., silicon and FN0603 alone and the two in combination) resulted in highly significant increases of the genus *Gibberella* (*p* < 0.01) and the highest abundance accounted for 14.84% of the inoculation with FN0603 in the present study. Some *Gibberella* could colonize wheat roots and significantly promote plant growth under water stress (Aletaha and Sinegani, 2020). In the present study, LEfSe assays showed that the abundance of these beneficial microorganisms was improved by FN0603 treatment, particularly in the combined treatment with silicon and FN0603, and were significantly correlated with FN0603 as biomarkers and were FN0603-specific biomarkers. The biomarkers significantly correlated with rhizosphere soil properties, plant physiology and wheat growth (Figures 4–6). Moreover, SEM analysis revealed that these biomarkers along with FN0603, directly and indirectly but positively affected the rhizosphere soil properties, plant physiology and wheat growth under saline-alkaline stress (Figure 7). Similarly, the inoculant BR indirectly associated with bacterial community, soil properties and plant growth by affecting BR specific biomarkers (Wang et al., 2021). The SEM also revealed that the rhizosphere soil properties and plant physiology was significantly correlated with wheat plant growth under saline-alkaline stress. Combined with the effects of the treatments on the rhizosphere soil properties, plant physiology and growth, we speculate that the combination of silicon and FN0603 promoted wheat plant growth by shaping the root endophytic microorganism community assembly.

## 5. Conclusion

**Figure 8:**
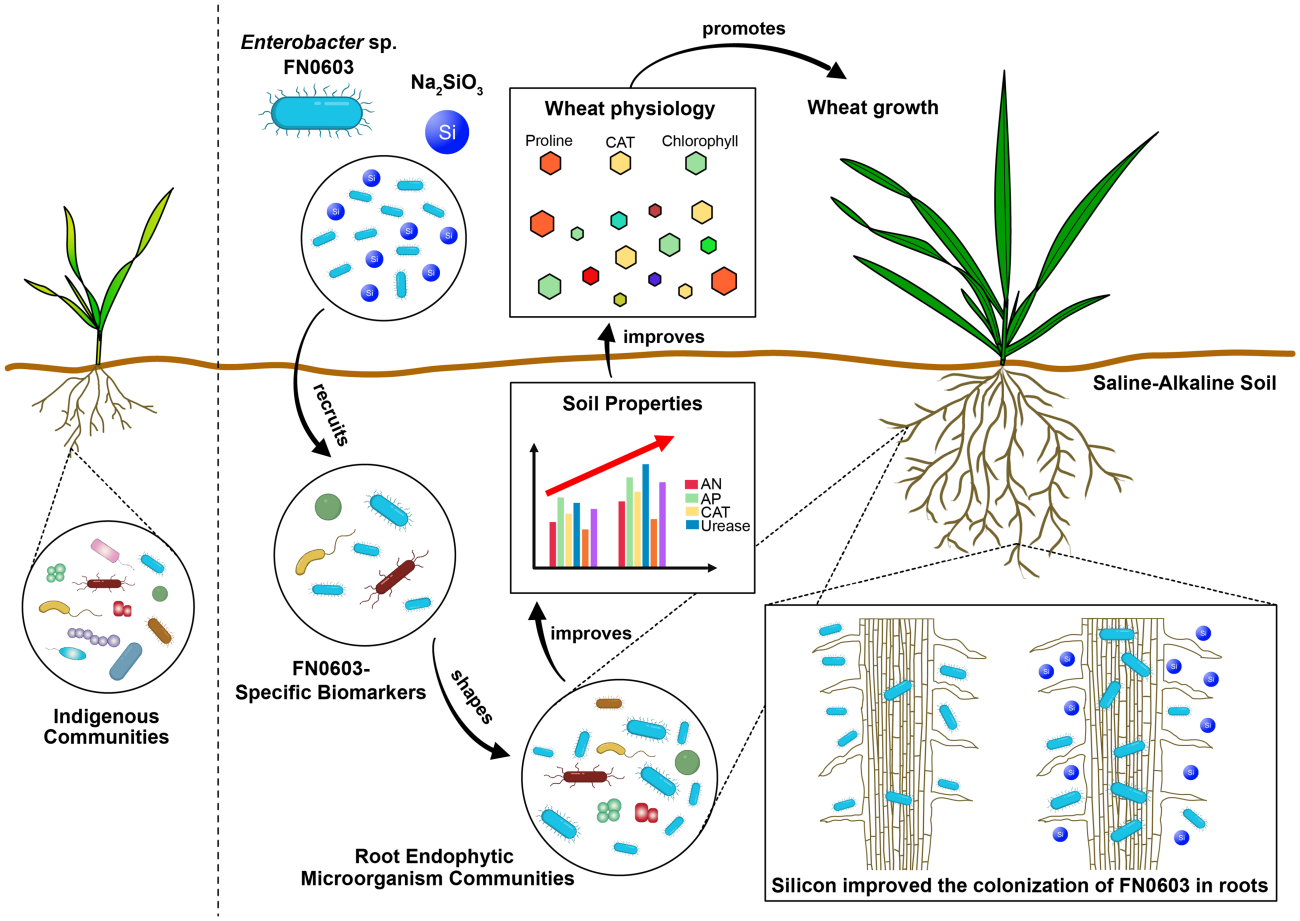
Mechanism of synergic wheat plant growth promotion by silicon and *Enterobacter* sp. FN0603 mitigate saline-alkaline stress.

Our work primarily studied the effects of combined silicon and PGPR treatment on plants under saline-alkaline stress. In this laboratory study, strain FN0603 was demonstrated to be an endophytic PGPR and to play a vital role in alleviating saline-alkaline stress in wheat, but silicon improved the colonization of FN0603 in roots and hence resulted in the synergic plant growth promotion effects when combined with FN0603. Mass colonization made FN0603 a biomarker, directed FN0603-specific biomarkers, and shaped the root endophytic microorganism community assembly. The combination of silicon and FN0603 most significantly promoted the wheat growth of under saline-alkaline by positively and remarkably affecting the rhizosphere soil properties, plant morphology and physiology, providing evidence that treatment with a combination of silicon and beneficial root endophytic microorganisms is an effective and promising approach to mitigate saline-alkaline stress on plants (Figure 8). However, further field trials are necessary to confirm these effects and mechanisms.

## Data availability statement

The datasets presented in this study can be found in online repositories. The names of the repository/repositories and accession number(s) can be found at: https://www.ncbi.nlm.nih.gov/genbank/, OP326194; https://www.ncbi.nlm.nih.gov/, PRJNA901197.

## Author contributions

FFX and FYF designed the study. FFX completed the experimental content, data analysis, and wrote the first draft of the manuscript. FYF revised the manuscript. YGL contributed some to community assays. XBW, YZG, and KT commented on the revision of the manuscript. All authors contributed to revising, read, and approved the submitted version.

## Funding

This work was financially supported by the National Natural Science Foundation of China (31960021) and Key Technology Research Project of Inner Mongolia Autonomous Region (2021GG0360).

## Conflict of interest

FFX is a student at Inner Mongolia Agricultural University.

The remaining authors declare that the research was conducted in the absence of any commercial or financial relationships that could be construed as a potential conflict of interest.

## Publisher’s note

All claims expressed in this article are solely those of the authors and do not necessarily represent those of their affiliated organizations, or those of the publisher, the editors and the reviewers. Any product that may be evaluated in this article, or claim that may be made by its manufacturer, is not guaranteed or endorsed by the publisher.

## Footnotes

^1^https://github.com/OpenGene/fastp, version 0.20.0

^2^http://www.cbcb.umd.edu/software/flash, version 1.2.7

^3^ http://huttenhower.sph.harvard.edu/LEfSe
