# Supplementary material for: Synergic mitigation of saline-alkaline stress in wheat plant by silicon and Enterobacter sp. FN0603
Source: Front Microbiol. 2023 Jan 16;13:1100232. doi: 10.3389/fmicb.2022.1100232 (PMC9885204; doi:10.3389/fmicb.2022.1100232)
Supplement: Supplementary file 1 [file Table_1.docx]

**Supplementary Table S1.** Plant growth-promoting traits of *Enterobacter* sp. FN0603

| Plant growth-promoting traits | Activity |
| --- | --- |
| Nitrogen fixation (nmol C_2_H_4_ h· mg pro) | 52.01 |
| Solubilization of insoluble phosphate (mg/L) | 29.26 |
| Siderophore production (%) | 60.53% |
| ACC deaminase (ng/mg pro) | 4.51 |
| Production of indole-3-Acetic Acid (IAA) (mg/L) | 19.72 |
| Production of biofilm (OD_590_) | 0.13 |
| Production of EPS (μg/mL) | 236.67 |
| Salinity (NaCl %) | 0-10(3) |
| pH | 4-12(10) |

**Supplementary Table S2.** Average tiller numbers per wheat

| Treatments |  | Tiller numbers | *p* < 0.05 | *p* < 0.01 |
| --- | --- | --- | --- | --- |
| NU |  | 0.40±0.00 | b | B |
| Si |  | 1.00±0.00 | b | B |
| FN |  | 1.20±0.45 | b | B |
| FN_Si |  | 3.00±0.00 | a | A |

**Supplementary Table S3.** Salt tolerance index of whole wheat dry weight

| Treatments | Salt tolerance index | *p* < 0.05 | *p* < 0.01 |
| --- | --- | --- | --- |
| Si/NU | 125.80±2.25 | d | D |
| FN/NU | 156.55±1.22 | b | B |
| FN_Si/NU | 182.08±2.40 | a | A |
| FN/Si | 124.48±2.81 | d | D |
| FN_Si/Si | 144.75±2.18 | c | C |
| FN_Si/FN | 116.32±1.84 | e | E |


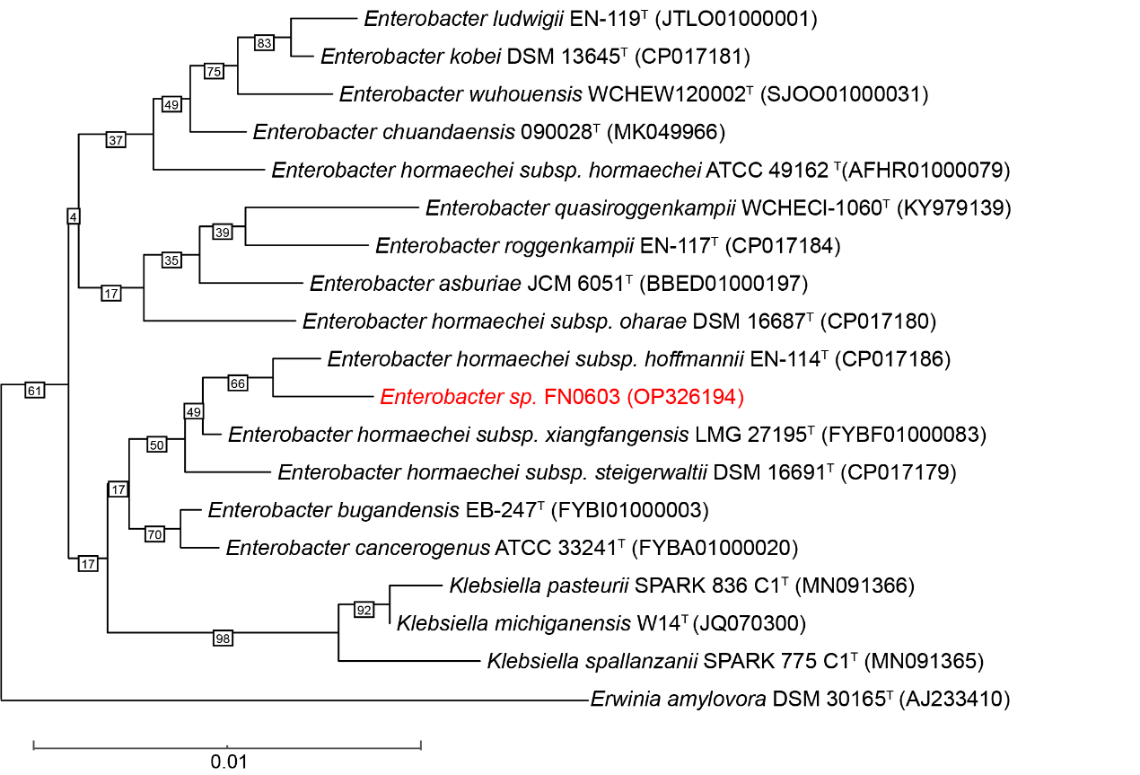
**Supplementary Figure S1.** A phylogenetic relationship of strain FN0603 identified on the basis of 16S rDNA gene sequences. The phylogenetic tree was constructed using Neighbor-joining method with 1000 replicates, numbers indicate 0.005 Knuc units.


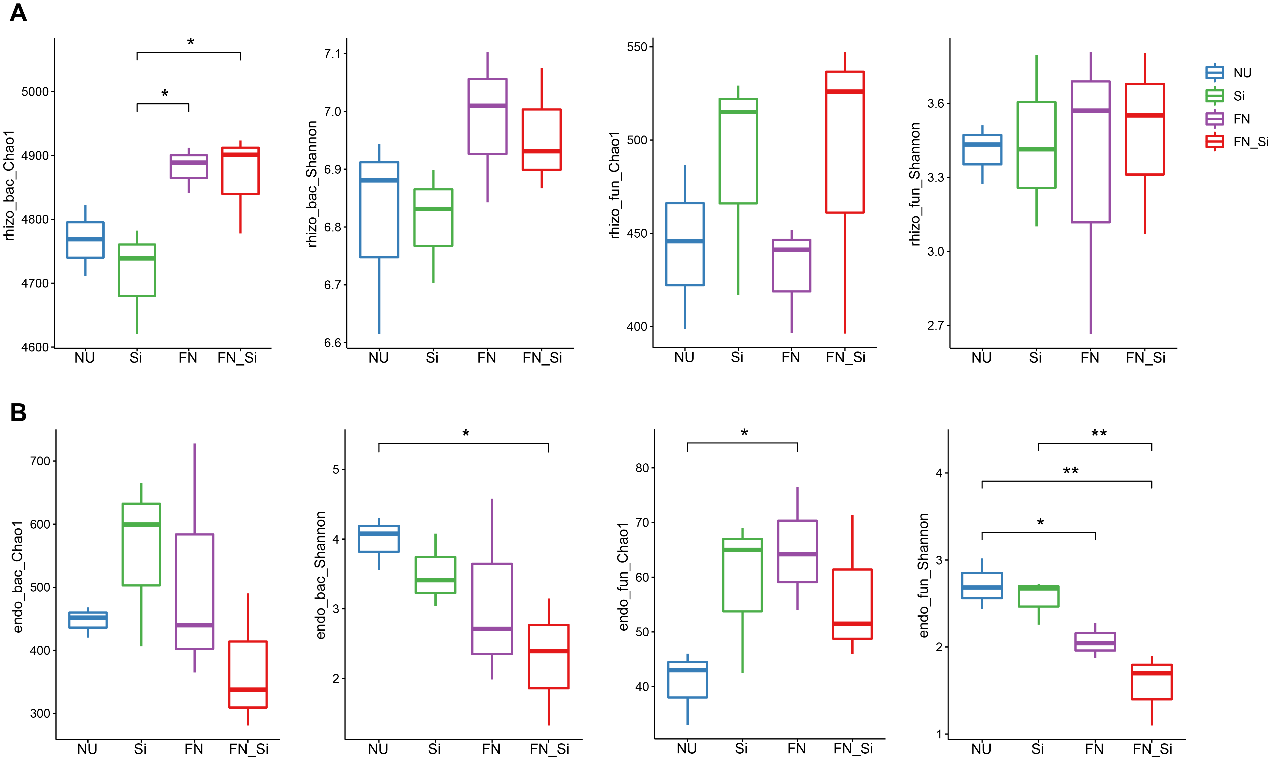


**Supplementary Figure S2.**  Chao 1 and Shannon indexes of *α*-Diversity of (A) rhizosphere microorganisms and (B) root endophytic microorganisms.


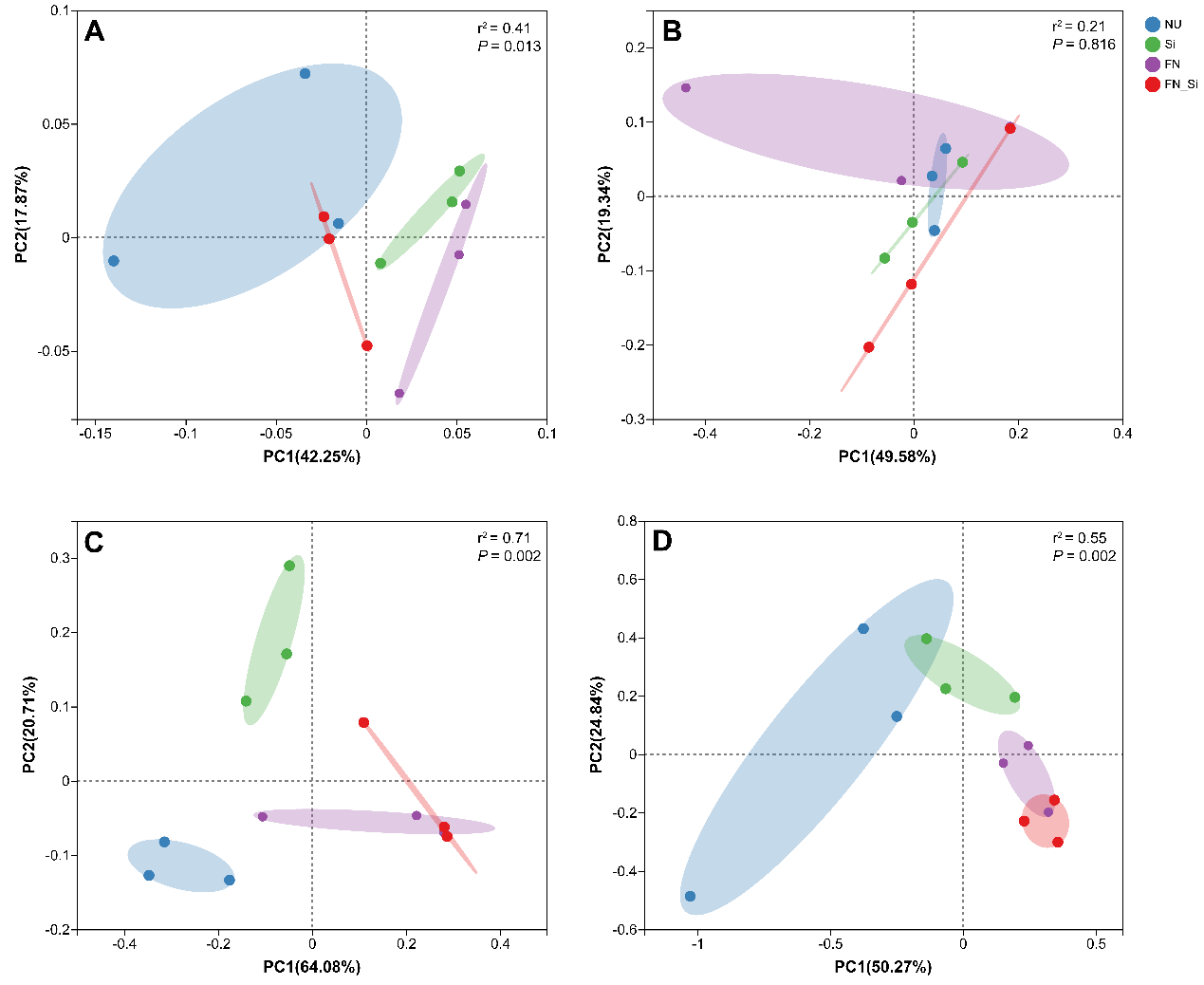


**Supplementary Figure S3.** The principal co-ordinates analysis (PCoA) of (A) rhizosphere bacterial communities, (B) rhizosphere fungal communities, (C) root endophytic bacterial communities and (D) root endophytic fungal communities based on Weighted-Unifrac distance.


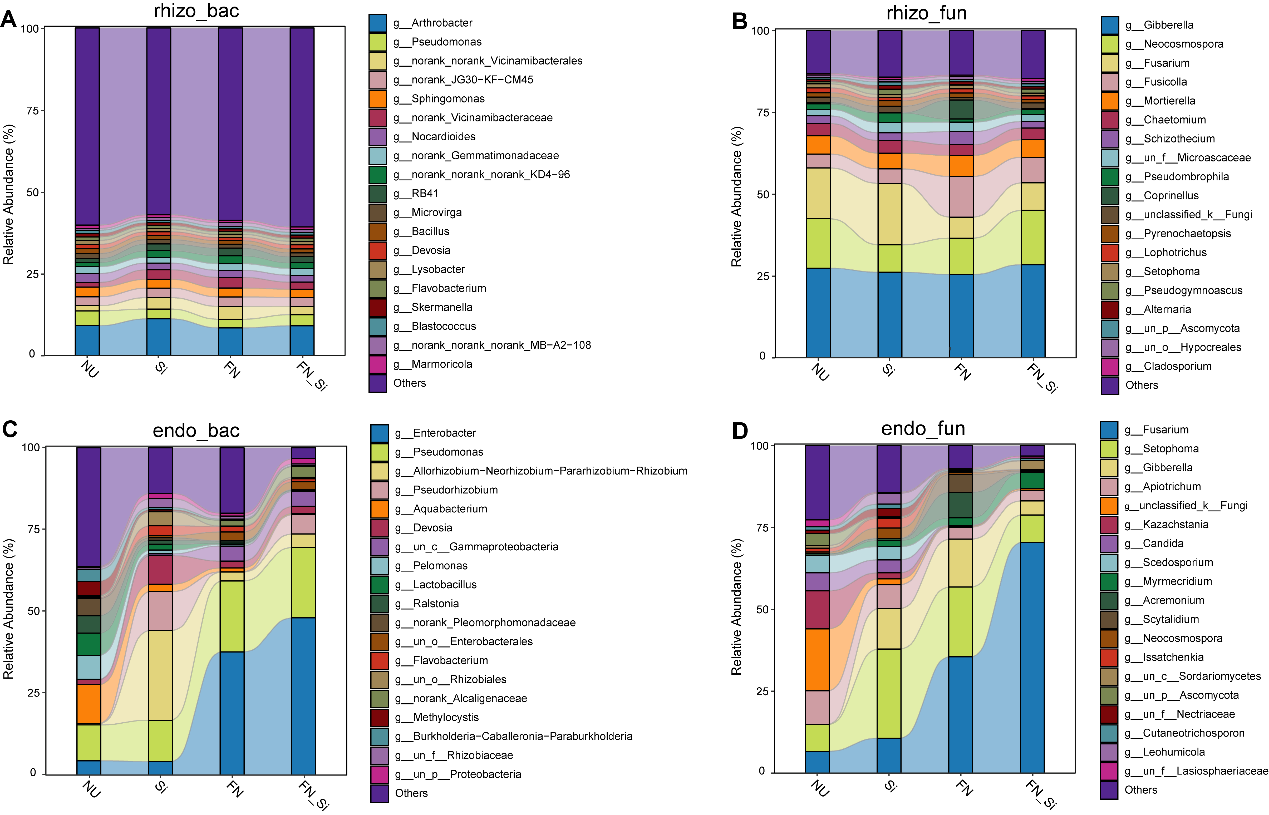
**Supplementary Figure S4.** (A) rhizosphere bacterial community, (B) rhizosphere fungal community, (C) root endophytic bacterial community and (D) root endophytic fungal community composition at genus level.


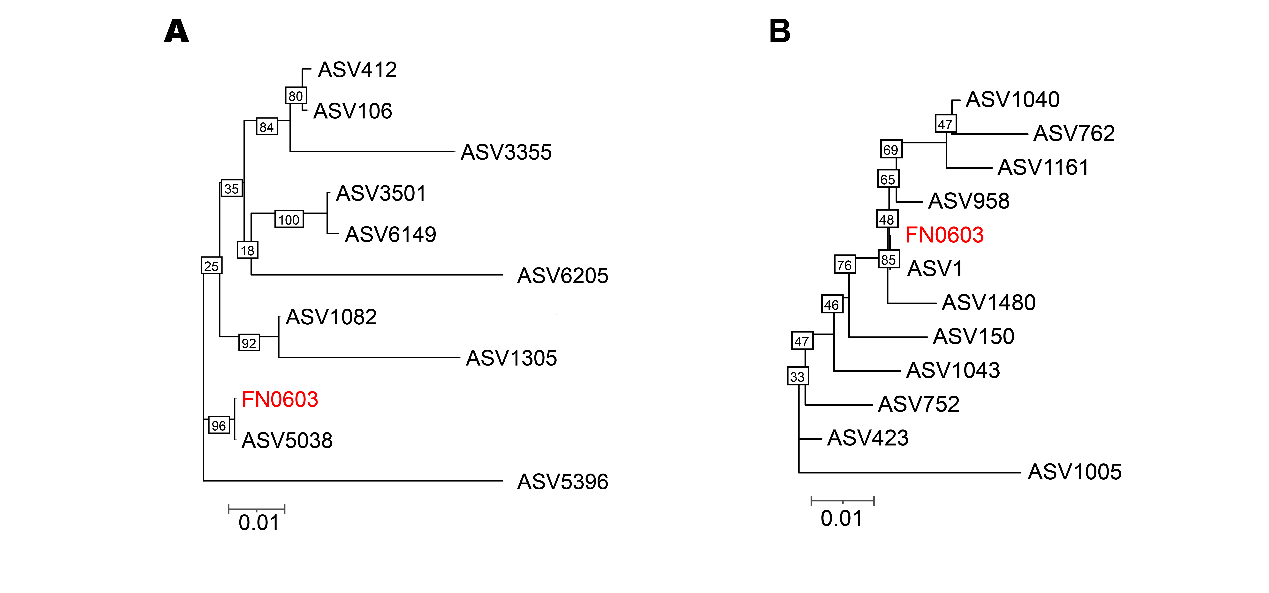


**Supplementary Figure S5.** (A) phylogenetic tree between strain FN0603 and ASV5038 in rhizosphere bacterial communities. (B) phylogenetic tree between strain FN0603 and ASV1 in endophytic bacterial communities.


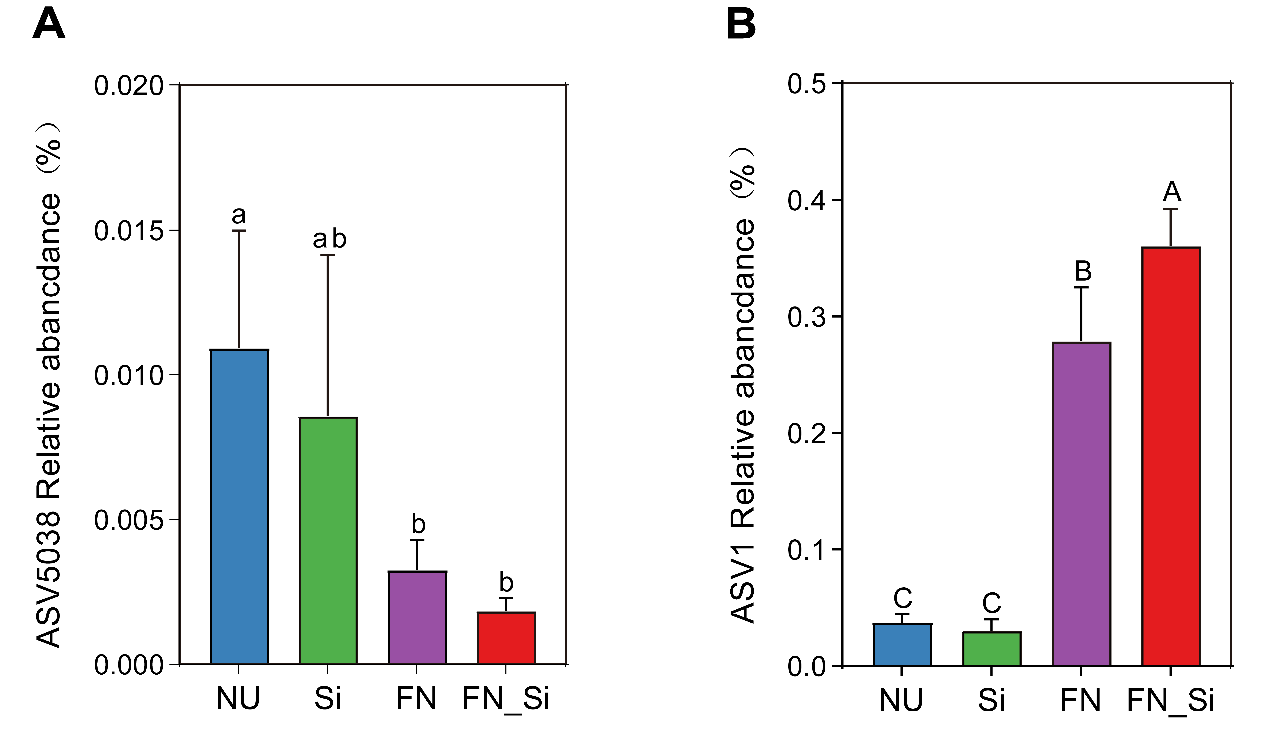
**Supplementary Figure S6.** Relative abundance of inoculated strain FN0603 in (A) rhizosphere bacterial communities and (B) root endophytic bacterial communities.


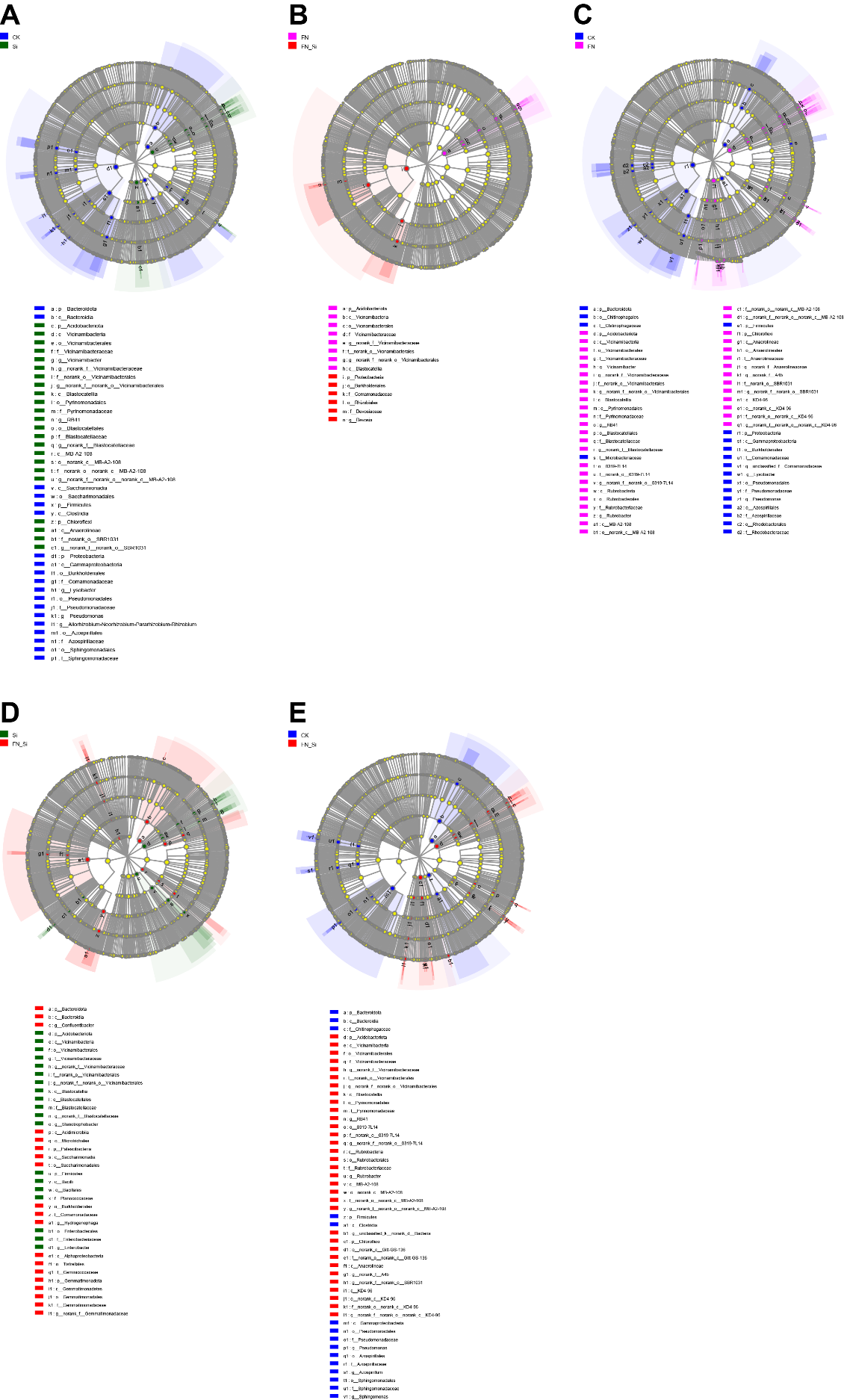
**Supplementary Figure S7.** Predicted Specific biomarkers on linear discriminant analysis effect size (LEfSe) of (A), (B), (C), (D) and (E) in rhizosphere bacterial communities with LDA score >3, *p* < 0.05.


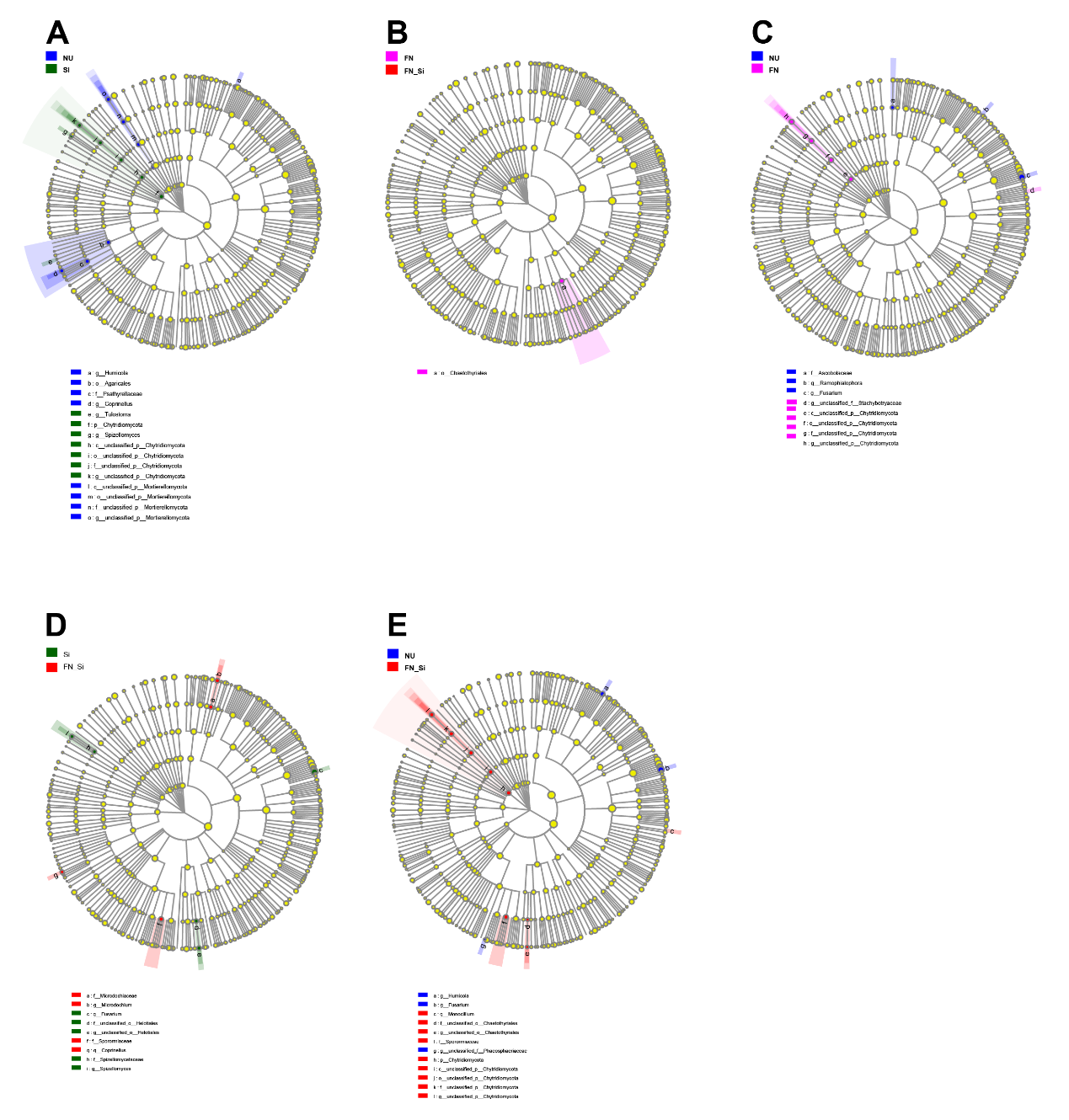
**Supplementary Figure S8.** Predicted Specific biomarkers on linear discriminant analysis effect size (LEfSe) of (A), (B), (C), (D) and (E) in rhizosphere fungal communities with LDA score >3, *p* < 0.05.


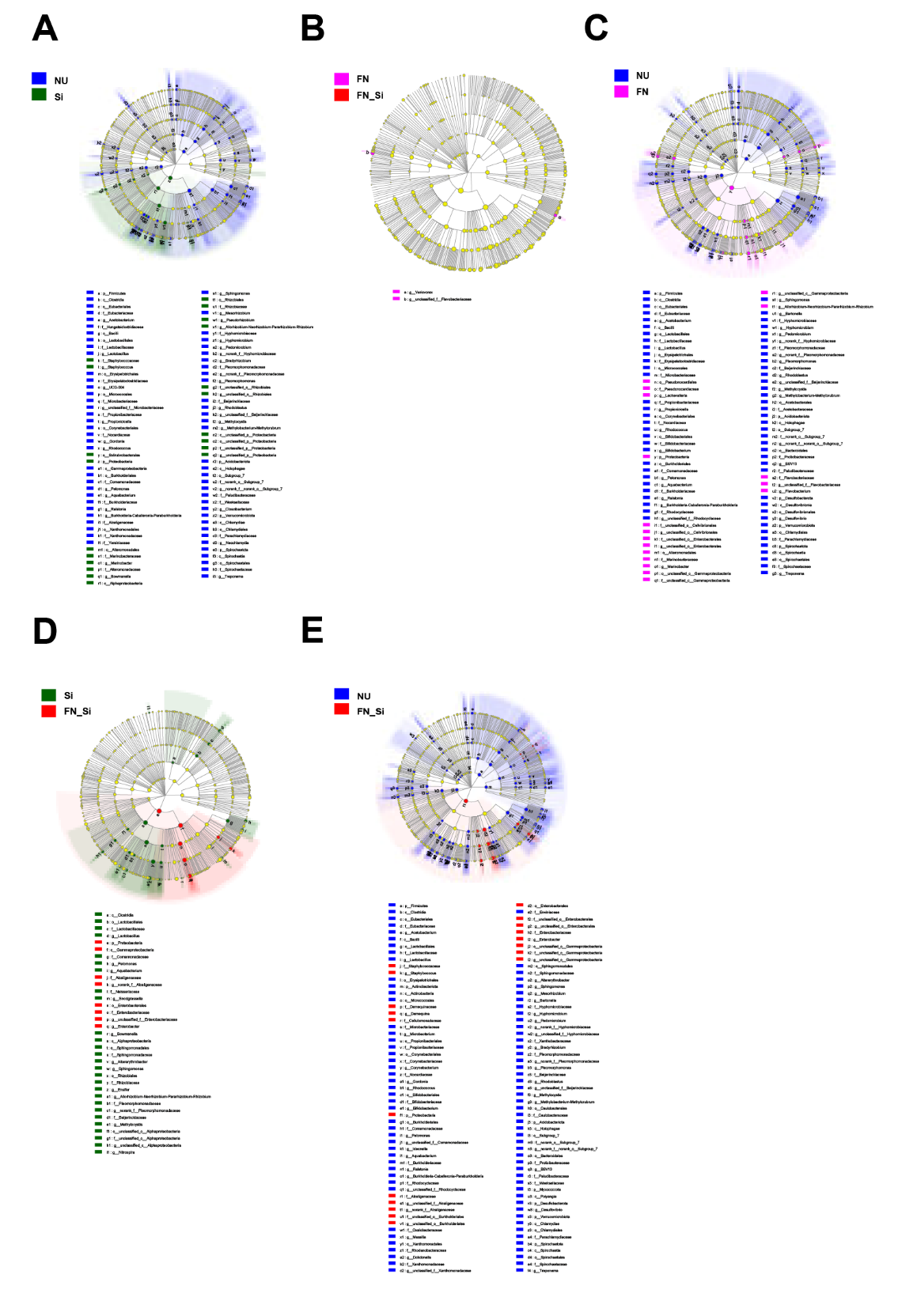
**Supplementary Figure S9.** Predicted Specific biomarkers on linear discriminant analysis effect size (LEfSe) of (A), (B), (C), (D) and (E) in endophytic bacterial communities with LDA score >3, *p* < 0.05.


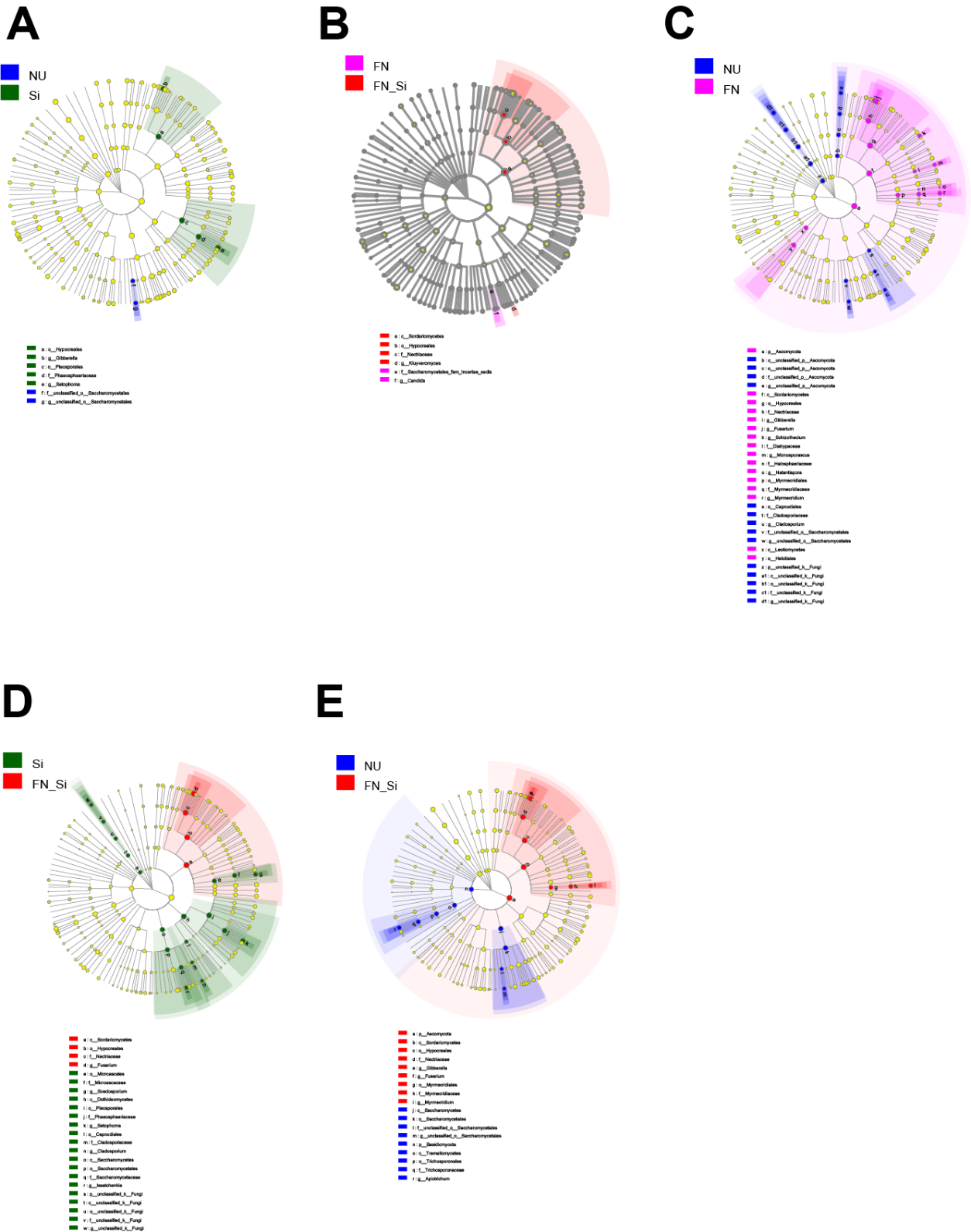
**Supplementary Figure S10.** Predicted Specific biomarkers on linear discriminant analysis effect size (LEfSe) of (A), (B), (C), (D) and (E) in endophytic fungal communities with LDA score >3, *p* < 0.05.


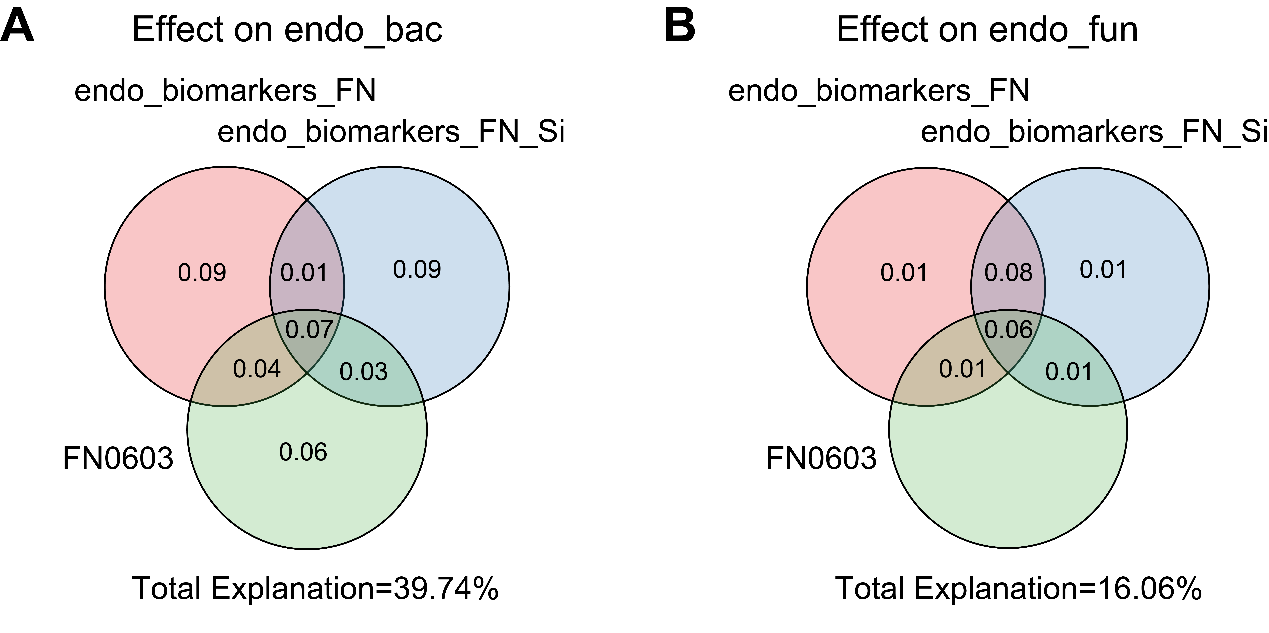
**Supplementary Figure S11.** Variation partitioning analysis (VPA) evaluated the explanatory degree of FN0603, endo_biomarkers_FN and endo_biomarkers_FN_Si to the variation of (A) root endophytic bacterial communities and (B) root endophytic fungal communities. All the specific biomarkers were FN0603-specific biomarkers.
